# Supplementary material for: Acute Rehabilitation following Traumatic anterior shoulder dISlocAtioN (ARTISAN): protocol for a multicentre randomised controlled trial
Source: BMJ Open. 2020 Nov 19;10(11):e040623. doi: 10.1136/bmjopen-2020-040623 (PMC7678365; doi:10.1136/bmjopen-2020-040623)
Supplement: Supplementary data [file bmjopen-2020-040623supp001.pdf]

WARWICK CLINICAL TRIALS UNIT

YTU York Trials Unit

<<INSERT LOCAL TRUST LOGO>>

NHS University Hospitals Coventry and Warwickshire NHS Trust

ARTISAN

Participant ID: | | | |

2. Main Consent Form

Please read each statement carefully and initial the box if you agree — Please *initial* box

1. I confirm that I have read and understood the information sheet (Version ___ Date ______) for the ARTISAN study. I have had the opportunity to consider the information, ask questions and have had these answered satisfactorily.	☐
2. I understand that my participation is voluntary and that I am free to withdraw at any time, without giving any reason, without my medical care or legal rights being affected.	☐
3. I understand that relevant sections of any of my medical notes and data collected during the study may be looked at by responsible individuals at regulatory authorities or from the NHS Trust, where it is relevant to my taking part in this research. I give permission for these individuals to have access to my records.	☐
4. I understand that appropriate personal identifying information will be collected, stored and used by Warwick Clinical Trials Unit to enable follow up of my health status. This is on the understanding that any information will be treated with the strictest security and confidentiality.	☐
5. I understand that the information held and maintained by the Health and Social Care Information Centre and other Central UK NHS bodies may be used to help contact me or provide information about my health status.	☐
6. I understand the information collected about me will be used to support other research in the future, and may be shared anonymously with other researchers.	☐
7. I agree to my GP being informed of my participation in the study.	☐
8. I agree to take part in the above study.	☐

The below are not mandatory but please let us know your preference.

9. I agree to being contacted about future research related to my shoulder dislocation.	Yes ☐	No ☐
10a. I agree to use the ARTISAN mobile App for follow-up data collection - if NO see 10b	Yes ☐	No ☐
10b. *If you have opted out of using the ARTISAN mobile app for follow up (10a), the method of follow-up will be via postal questionnaires, do you consent to text message reminders by the ARTSAN study*	Yes ☐	No ☐
11. I agree to my session being observed and audio recorded to monitor quality.	Yes ☐	No ☐

Patient Name	Signature	Date (DD/MMM/YYYY)
Name of person taking consent	Signature	Date (DD/MMM/YYYY)

Original copy to be kept in the ARTISAN site file, one copy to be given to the patient and one copy to be kept in the participants medical notes.

This project is funded by the NIHR HTA programme (Ref: 16/167/56).

FUNDED BY NIHR | National Institute for Health Research

ARTISAN Main Consent Form v5.0 | 15.04.2020 IRAS Project ID: 245863
