# Supplementary material for: Acute Rehabilitation following Traumatic anterior shoulder dISlocAtioN (ARTISAN): protocol for a multicentre randomised controlled trial
Source: BMJ Open. 2020 Nov 19;10(11):e040623. doi: 10.1136/bmjopen-2020-040623 (PMC7678365; doi:10.1136/bmjopen-2020-040623)
Supplement: Author's manuscript [file bmjopen-2020-040623.draft_revisions.pdf]

BMJ Open

BMJ Open is committed to open peer review. As part of this commitment we make the peer review history of every article we publish publicly available.

When an article is published we post the peer reviewers' comments and the authors' responses online. We also post the versions of the paper that were used during peer review. These are the versions that the peer review comments apply to.

The versions of the paper that follow are the versions that were submitted during the peer review process. They are not the versions of record or the final published versions. They should not be cited or distributed as the published version of this manuscript.

BMJ Open is an open access journal and the full, final, typeset and author-corrected version of record of the manuscript is available on our site with no access controls, subscription charges or pay-per-view fees (http://bmjopen.bmj.com).

If you have any questions on BMJ Open's open peer review process please email info.bmjopen@bmj.com

BMJ Open

Acute Rehabilitation following Traumatic anterior shoulder dISlocAtioN (ARTISAN): Protocol for a Multi Centre Randomised Controlled Trial

Journal:	*BMJ Open*
Manuscript ID	bmjopen-2020-040623
Article Type:	Protocol
Date Submitted by the Author:	18-May-2020

Complete List of Authors:	Kearney, Rebecca; University of Warwick, Warwick Clinical Trials Unit<br>Dhanjal, Gurmit; University of Warwick, Warwick Clinical Trials Unit<br>Parsons, Nicholas; University of Warwick, Warwick Medical School<br>Ellard, David; University of Warwick, Clinical Trials Unit;<br>Parsons, Helen; University of Warwick, Division of Health Sciences<br>Haque, Aminul; University of Warwick, Warwick Clinical Trials Unit<br>Karasouli, Eleni; University of Warwick, Warwick Clinical Trials Unit<br>Mason, James; University of Warwick, Warwick Medical School<br>Nwankwo, Henry; University of Warwick, Warwick Clinical Trials Unit<br>Brown, Jaclyn; University of Warwick, Clinical Trials Unit; University of Warwick Clinical Sciences Research Institute, CTU<br>Liew, Zi Heng; University of Warwick, Warwick Clinical Trials Unit, Warwick Medical School<br>Drew, Stephen; University Hospitals Coventry and Warwickshire NHS Trust, Trauma and Orthopaedics<br>Modi, Chetan; University Hospitals Coventry and Warwickshire NHS Trust, Trauma and Orthopaedics<br>Bush, Howard; University Hospitals Coventry and Warwickshire NHS Trust, Trauma and Orthopaedics<br>Torgerson, David; The University of York,<br>Underwood, Martin; Warwick University, Warwick Medical School

Keywords:	Shoulder < ORTHOPAEDIC & TRAUMA SURGERY, Rehabilitation medicine < INTERNAL MEDICINE, Clinical trials < THERAPEUTICS

SCHOLARONE™ Manuscripts

I, the Submitting Author has the right to grant and does grant on behalf of all authors of the Work (as defined in the below author licence), an exclusive licence and/or a non-exclusive licence for contributions from authors who are: i) UK Crown employees; ii) where BMJ has agreed a CC-BY licence shall apply, and/or iii) in accordance with the terms applicable for US Federal Government officers or employees acting as part of their official duties; on a worldwide, perpetual, irrevocable, royalty-free basis to BMJ Publishing Group Ltd ("BMJ") its licensees and where the relevant Journal is co-owned by BMJ to the co-owners of the Journal, to publish the Work in this journal and any other BMJ products and to exploit all rights, as set out in our licence.

The Submitting Author accepts and understands that any supply made under these terms is made by BMJ to the Submitting Author unless you are acting as an employee on behalf of your employer or a postgraduate student of an affiliated institution which is paying any applicable article publishing charge ("APC") for Open Access articles. Where the Submitting Author wishes to make the Work available on an Open Access basis (and intends to pay the relevant APC), the terms of reuse of such Open Access shall be governed by a Creative Commons licence – details of these licences and which Creative Commons licence will apply to this Work are set out in our licence referred to above.

Other than as permitted in any relevant BMJ Author's Self Archiving Policies, I confirm this Work has not been accepted for publication elsewhere, is not being considered for publication elsewhere and does not duplicate material already published. I confirm all authors consent to publication of this Work and authorise the granting of this licence.

For peer review only

TITLE PAGE

Title

Acute Rehabilitation following Traumatic anterior shoulder dISlocAtioN (ARTISAN): Protocol for a Multi Centre Randomised Controlled Trial

Corresponding author

Rebecca S Kearney: Professor, University of Warwick, Clinical Trials Unit, Gibbet Hill Road, Coventry, CV4 7AL & University Hospitals Coventry & Warwickshire NHS Trust, Trauma and Orthopaedics, Clifford Bridge Road, Coventry, CV2 2DX; R.S.Kearney@warwick.ac.uk; 02476 573156

Authors

- Gurmit Dhanjal: Trial Manager, University of Warwick, Warwick Medical School, Gibbet Hill Road, Coventry, CV4 7AL (Gurmit.Dhanjal@warwick.ac.uk)
- Nick Parsons: Associate Professor, University of Warwick, Warwick Medical School, Gibbet Hill Road, Coventry, CV4 7AL (Nick.Parsons@warwick.ac.uk)
- David Ellard: Associate Professor, University of Warwick, Clinical Trials Unit, Gibbet Hill Road, Coventry, CV4 7AL (D.R.Ellard@warwick.ac.uk)
- Helen Parsons: Senior Research Fellow, University of Warwick, Clinical Trials Unit, Gibbet Hill Road, Coventry, CV4 7AL (H.Parsons@warwick.ac.uk)
- Aminul Haque: Research Associate, University of Warwick, Clinical Trials Unit, Gibbet Hill Road, Coventry, CV4 7AL (Aminul.Haque.1@warwick.ac.uk)
- Eleni Karasouli: Senior Research Fellow, University of Warwick, Clinical Trials Unit, Gibbet Hill Road, Coventry, CV4 7AL (E.Karasouli@warwick.ac.uk)
- James Mason: Professor, University of Warwick, Clinical Trials Unit, Gibbet Hill Road, Coventry, CV4 7AL (J.Mason@warwick.ac.uk)
- Henry Nwankwo: Research Associate, University of Warwick, Clinical Trials Unit, Gibbet Hill Road, Coventry, CV4 7AL (Henry.Nwankwo@warwick.ac.uk)
- Jaclyn Brown: Deputy Head of Operations, University of Warwick, Clinical Trials Unit, Gibbet Hill Road, Coventry, CV4 7AL (J.Brown.10@warwick.ac.uk)

For peer review only - http://bmjopen.bmj.com/site/about/guidelines.xhtml

- ZiHeng Liew: Research Physiotherapist, University of Warwick, Clinical Trials Unit, Gibbet Hill Road, Coventry, CV4 7AL (Zi.Liew@warwick.ac.uk)
- Stephen Drew: Consultant Orthopaedic Surgeon, University Hospitals Coventry & Warwickshire NHS Trust, Trauma and Orthopaedics, Clifford Bridge Road, Coventry, CV2 2DX (Email: Steven.Drew@uhcw.nhs.uk)
- Chetan Modi: Consultant Orthopaedic Surgeon, University Hospitals Coventry & Warwickshire NHS Trust, Trauma and Orthopaedics, Clifford Bridge Road, Coventry, CV2 2DX (Email: Chetan.Modi@uhcw.nhs.uk)
- Howard Bush: Physiotherapist, University Hospitals Coventry & Warwickshire NHS Trust, Trauma and Orthopaedics, Clifford Bridge Road, Coventry, CV2 2DX (Email: Howard.Bush@uhcw.nhs.uk)
- David Torgerson: Professor, University of York, York Trials Unit, Department of Health Sciences, ARRC Building, Heslington, York, YO10 5DD (david.torgerson@york.ac.uk)
- Martin Underwood: Professor, University of Warwick, Clinical Trials Unit, Gibbet Hill Road, Coventry, CV4 7AL & University Hospitals Coventry & Warwickshire NHS Trust, Trauma and Orthopaedics, Clifford Bridge Road, Coventry, CV2 2DX (M.Underwood@warwick.ac.uk)

Word count: 3885

For peer review

ABSTRACT

Introduction

First time Traumatic Anterior Shoulder Dislocation (TASD) is predominantly managed non-operatively. People sustaining TASD have ongoing pain, disability, and substantial morbidity linked to high recurrence rates and subsequent need for repeated episodes of management. There are no published RCTs comparing non-operative management strategies to ascertain what rehabilitation strategy after TASD is cost-effective.

Methods and analysis

In this multi-centre adaptive RCT, with internal pilot, adults with a radiologically confirmed first time TASD treated non-surgically will be screened at a minimum of 30 sites. People with neurovascular complications, bilateral dislocations, or are unable to attend physiotherapy will be excluded.

For peer review only - http://bmjopen.bmj.com/site/about/guidelines.xhtml

Randomisation will be on a 1:1 treatment allocation, stratified by age, hand dominance and site. Participants will receive a single session of advice; or a single session of advice plus offer of further physiotherapy (maximum six sessions). The primary analysis will be the difference in Oxford Shoulder Instability Score (OSIS) at six months. A sample size of a minimum of 478 participants will allow us to show a four point difference with 90% power.

An embedded qualitative study will explore the participants' experiences of the trial interventions.

Ethics, registration and dissemination

Funded by NIHR HTA (16/167/56), 01/06/18; National Research Ethic Committee approved (18/WA/0236), 26/07/18. Registered on the International Standard Randomised Controlled Trial Number registry (ISRCTN63184243), 07/09/18. First site opened 05/11/18 and final results will be updated on trial registries and submitted to a peer-reviewed journal and will inform rehabilitation strategies after a TASD. SWAT funded by MRC (MR/R013748/1), 01/05/19; registered on the MRC-HTMR All-Ireland Hub (reference number SWAT 121).

Keywords

Shoulder dislocation, rehabilitation, physiotherapy, adaptive design

ARTICLE SUMMARY: STRENGHTS AND LIMITATIONS OF THIS STUDY

- UK wide trial across a minimum of 30 sites to optimise external validity.
- Primary outcome measure is patient-centred.
- The main limitation is that the intervention could not be blinded.

MAIN TEXT

Introduction

The shoulder is the most frequently dislocated joint; occurring in 8.2 to 23.9 per 100,000 people per year [1]. Traumatic Anterior Shoulder Dislocations (TASD) occur when excessive forces during a

For peer review only - http://bmjopen.bmj.com/site/about/guidelines.xhtml

traumatic event displace the humeral head frontwards, out of the shoulder socket, resulting in the joint surfaces completely losing contact [1-3]. They predominantly occur in males under 25 years during high impact events and females over 80 years during low impact events [1].

People sustaining TASD often have ongoing pain, disability, and morbidity linked to re-dislocation and subsequent need for repeated management [1-3]. Rehabilitation may reduce these events and restore a functional, painless shoulder through restoration of movement and retraining muscles [2]. However, a Cochrane review has found no evidence to support this [2]; Dutch national guidelines explicitly do not refer to physiotherapy [3]; and UK guidelines cite referral *'may be helpful'* [1].

This protocol outlines the research to provide evidence regarding the nature and extent of what physiotherapy is required for the management of patients following TASD with an embedded 'Study Within A Trial (SWAT)' to compare two strategies for retention of participants in RCTs.

Research questions

RCT Research question

In adults with first time TASD managed non-operatively does a single session of advice and course of physiotherapy improve the Oxford Shoulder Instability Score (OSIS) by a minimum of four additional points at six months when compared to a single session of advice only?

SWAT Research question

Does making a courtesy introductory telephone call to recruited participants improve response rates to six month follow-up questionnaires compared with a written card with similar information?

Objectives

RCT Primary objective

To quantify and draw inferences on observed differences in the OSIS score between a single session of advice and physiotherapy with a single session of advice only, for adults with first time TASD managed non-operatively, six months post randomisation.

For peer review only - http://bmjopen.bmj.com/site/about/guidelines.xhtml

RCT Secondary objectives

- To estimate comparative cost-effectiveness (cost/QALY) of the two trial treatments, from an NHS and personal social services perspective.
- To determine the difference in complication rate (e.g. shoulder re-dislocation) in the first 12 months between the trial treatment groups.
- To quantify and draw inferences between the functional status (OSIS) of the trial treatment groups at six weeks, three and 12 months.
- To quantify and draw inferences on observed differences in the functional status (QuickDASH) of between the trial treatment groups at six weeks, three, six and 12 months.
- To quantify and draw inferences on observed differences of health related quality of life (EQ-5D-5L) between the trial treatment groups at six weeks, three, six and 12 months.
- To qualitatively explore participants' experiences of receiving the trial treatments and facilitators and obstacles to adhering to them.

SWAT Primary objective

Evaluate the impact of making a courtesy call to newly recruited participants in the ARTISAN trial on response rates to six month follow-up questionnaires compared with a written card with similar information.

SWAT Secondary objectives

- Time to response to the questionnaires at each time points.
- Response rates at six weeks, three, six and twelve months.
- Completeness of responses.
- Number of reminder notices required.
- Cost of intervention per participant.

Methods and analysis

Study design

For peer review only - http://bmjopen.bmj.com/site/about/guidelines.xhtml

Multi-centre, adaptive, randomised controlled trial with internal pilot and SWAT

Outcome measures

Outcome measures will be collected at baseline (pre randomisation) at the recruiting site and then at six weeks, three, six and twelve months centrally from Warwick Clinical Trials Unit (WCTU) by postal questionnaire, mobile application, telephone or email.

RCT Primary outcome

OSIS: Self-completed outcome measure containing 12 questions (0-4 points each), with possible scores from 0 (best function) to 48 (worst function) [4, 5]. Questions relate to activities of daily living particularly relevant to patients exhibiting shoulder instability and has been designed to assess outcome of therapy.

RCT Secondary outcomes

QuickDASH: Self-completed shortened version of the Disabilities of the Arm, Shoulder and Hand (DASH) questionnaire. Uses 11 items to measure physical function and symptoms in people with any or multiple musculoskeletal disorders of the upper limb. Designed to help describe the disability experienced by people with upper-limb disorders and monitor changes in symptoms and function over time [6].

EQ-5D-5L: Health-related quality of life measure consisting of five dimensions, each with 5-levels of response. Each combination of answers is converted into a health utility score. Gives a single preference based index value for health status that can be used for broader cost-effectiveness comparative purposes [7].

Complications: Complications will be defined into three categories:
a) Pre-defined complications directly related to the interventions.
b) Pre-defined complications directly caused by the primary TASD event not identified by the initial assessing clinician, but subsequently identified.
c) Complications not related to the intervention or TASD event and will subsequently not be formally analysed or reported.

For peer review only

For peer review only - http://bmjopen.bmj.com/site/about/guidelines.xhtml

Resource use questionnaires: Primary health-economic analysis will concentrate on direct intervention and healthcare/personal social services costs, while wider impact (societal) costs will be included within the sensitivity analyses.

Embedded qualitative interviews: A purposive sample, up to 50 participants, informed by treatment allocation, gender, age, and outcome will be invited for one interview, to explore the participant experience of receiving the interventions and facilitators and obstacles to adhering to them.

SWAT Primary outcome

Response rate: Proportion of participants who return the questionnaire.

SWAT Secondary outcomes

Time to response: Date of first sending questionnaire to date of questionnaire received by study team.

Completeness of responses: Number of missing items per questionnaire.

Number of reminder notices: Number of notices administered by the trial team.

Cost of intervention: Cost of phone call or postcard per participant.

Sample size

RCT Sample size

A worthwhile difference from this intervention is 4 points on the OSIS [8, 9]. The standard deviation (SD) of the OSIS six months after injury is 10 points [8, 9]. However, the literature has predominantly included a young population, given that we will recruit a wider range of ages a larger SD is expected, therefore an SD of 12 is more likely. To show a four point difference at 5% significance level with 90% power allowing a margin of 20% loss during follow-up needs a minimum of 478 participants [5, 9].

For peer review only - http://bmjopen.bmj.com/site/about/guidelines.xhtml

SWAT Sample size

The sample size for the SWAT is limited by the host trial sample size. The SWAT opened to recruitment after the internal pilot (50 participants), therefore approximately 428 participants (214 per group) are expected and would provide 90% power and 5% significance to detect differences in retention rates of approximately 11%.

Screening and eligibility

RCT Screening and eligibility

Adults presenting at trial sites (NHS hospitals) with a first-time TASD, confirmed radiologically, managed non-operatively, will be identified as potential participants. The site team will undertake eligibility checks and details will be entered on a monthly screening log.

Participants will be excluded if they have significant neurovascular complications; bilateral dislocations; have been randomised previously; unable to adhere to trial procedures, or unable attend physiotherapy within six weeks of injury.

All eligible participants will be provided with verbal and written information about the study. If eligible and willing to join the study, consent procedures will be completed followed by collection of baseline demographic data, pre and post injury functional outcomes.

SWAT screening and eligibility

Participants recruited into ARTISAN, who consent to being contacted by multiple methods, will be eligible for the SWAT.

Randomisation

RCT Randomisation

For peer review only - http://bmjopen.bmj.com/site/about/guidelines.xhtml

Participants will be randomised at the end of the initial advice session via a web based system. Allocation concealment will be maintained by an independent randomisation team at WCTU, who will be responsible for generation of the sequence and will have no role in the allocation of participants. The treatment group will be allocated by computer using a minimisation algorithm with a random element and stratification by participant age (39 years old and under and 40 years old and over), hand dominance and recruiting site.

SWAT randomisation

Minimisation with a random factor will be used to avoid imbalance between the SWAT intervention arms. The allocation ratio will be 1:1 and stratified by the ARTISAN allocation arm. Allocation concealment will be maintained by an independent randomisation team who will be responsible for generation of the sequence and have no role in the allocation of participants.

Post randomisation withdrawals

Participants may discontinue at any time without prejudice. Unless a participant explicitly withdraws consent, data will be collected until the end of the trial. For participants explicitly withdrawing consent for follow up procedures, trial data obtained up until the point of withdrawal will be included. Participants will have the option to withdraw from the trial-related questionnaires, but continue to provide routine NHS data for trial purposes. Participants who withdraw will not be replaced. Participants may be withdrawn from the trial at the discretion of the investigator and/or Trial Steering Committee for safety concerns.

Interventions

RCT Interventions

The interventions will be reported in line with the TIDIER and CERT checklists [10, 11]. All participating centres will receive an initial training session from the trial team. Following this, a lead physiotherapist at each site will be identified to complete subsequent training of additional physiotherapists. This training will be supported with web-based materials and a trial intervention manual.

For peer review only - http://bmjopen.bmj.com/site/about/guidelines.xhtml

All participants will receive a period of initial immobilisation [1]. At this time participants will be provided with a web-link to Phase 1 of the ARTISAN advice materials and provided with a paper based booklet version covering:

- What has happened to me?
- What can go wrong?
- How do I stop this happening again?
- How long do I have to wear my sling?
- Should I move my arm?
- How do I control my pain?
- When can I return to usual activities?
- What if something goes wrong?

All participants will subsequently receive an appointment for a physiotherapy advice session within six weeks of injury. At the first appointment, all participants will receive a single session of advice to aid self-management, lasting up to one hour and administered by an ARTISAN trained physiotherapist. Following routine assessment, the physiotherapist will deliver the above core set of intervention components and in addition:

- Points of contact if complications occur or expected recovery times are not achieved.
- A core set of progressive phase 2 range of movement exercises and what they aim to achieve.
- Enhancing self-management behaviours through the addition of goal setting, exercise planning and diaries.

The physiotherapist will provide details of web based materials, which will include all the core components above in written and video format, and will include a dedicated area for participants to set goals and keep diaries. The physiotherapist will also inform the participants that the website resources contain phase 3 progressive strengthening exercises and what they aim to achieve and later stage phase 4 progression to aid return to sports. Participants will be offered paper based alternatives.

Following completion of the first physiotherapy appointment the participant will be randomised, allocating them to this advice session alone or to this advice session plus the offer of additional physiotherapy.

For peer review only - http://bmjopen.bmj.com/site/about/guidelines.xhtml

For peer review only

Participants randomised to the advice session only, will only receive this physiotherapy intervention. However, if they are experiencing ongoing issues which are not improving, as part of the control intervention, the advice is for the participant to contact the clinic they attended or see their GP. If a participant follows this advice and self-initiates further physiotherapy this will not be deemed a deviation from the protocol. However, if someone other than the participant initiates further physiotherapy (another clinician) then this will be deemed a protocol deviation.

Participants randomised to receive additional physiotherapy will be offered of at least one additional physiotherapy session. Each additional session will last for up to 30 minutes, over a maximum duration of four months from date of randomisation. The course of physiotherapy will involve teaching and supervising the 'core set' of progressive exercises offered to the control arm in addition to being able to tailor treatment as per their usual practice.

SWAT Interventions

Participants will be randomised to receive either:

1) An introductory call within two weeks of being randomised to include thanking participants; reminding them of their valuable contribution and that they will be contacted at six weeks and three, six and twelve months; informing them of when the trial results are expected and to contact the ARTISAN team if they have queries.

2) A postcard-sized hand written card, with similar content as above, signed by the Chief Investigator and Trial Manager, posted to participants within one week of being randomised.

Fidelity and quality assurance of interventions

The trial team will implement a standardised approach of evaluating fidelity [12] of direct observations and audio recordings (twice annually); and self-reporting of the trial interventions. Any issues will be discussed by the trial management group (TMG), who will recommend appropriate action. If issues are not resolved following recommendations they will be escalated.

During site quality assurance visits the research teams will also be asked to provide radiological confirmation of the participants initial shoulder dislocation. If evidence is not found, participants will

For peer review only - http://bmjopen.bmj.com/site/about/guidelines.xhtml

be withdrawn from the main trial sample, but will continue follow up procures and retained as part of a pre-planned sensitivity analysis.

Blinding

As the type of rehabilitation used will be clear to the participant, they cannot be blind to treatment. The treating clinician will also not be blind, but will take no part in follow up processes, these will be managed centrally from the Warwick CTU to reduce the assessment bias.

Adverse event management

Serious adverse events (SAEs) will be entered onto the SAE reporting form and reported to the central study team. SAEs that may be expected will be pre-defined and recorded on the participant's follow up questionnaire. SAEs that may be expected as part of a TASD are: damage to nerve or blood vessels, fractures, re-dislocation, torn ligaments or muscles, persistent exacerbation of shoulder pain, restriction of range of movement, adhesive capsulitis (frozen shoulder) and persistent instability. All SAEs will be reported to the sponsor (Warwick University and University Hospitals Coventry and Warwickshire), ethics committee and oversight committees.

End of trial

The trial will end when all participants have completed their 12-month follow-up. The trial will be stopped prematurely if mandated by the Ethics Committee; following recommendations from relevant oversight committees; or funding ceases.

Trial oversight

The TMG will meet monthly and report to the independent Trail Steering Committee (TSC) and Data Monitoring and Safety Committee (DMSC). The TSC and DMSC will hold meetings annually and be responsible for approval of the protocol, monitoring and supervising progress.

Patient and Public Involvement (PPI)

For peer review only - http://bmjopen.bmj.com/site/about/guidelines.xhtml

The clinical team consulted with patients when developing this project and subsequently we had a meeting with a group representing our target participants. We discussed experiences and expectations of services and plans for the trial. Service users' perspectives were key in the development of this protocol. PPI membership is represented on TMG and TSC committees and have reviewed all study materials. PPI members of the team will contribute to dissemination on trial completion.

Analysis

RCT Statistical Analysis

The internal pilot will inform and test recruitment strategies for the main trial in 12 sites over six months. A recruitment rate of one participant per month per centre was estimated for the six month pilot phase, with 50 participants in total. The decision to progress to the main trial will be made based on pre-defined progression criteria. These randomised participants will be retained in the final analysis.

A detailed statistical analysis plan (SAP) which will be agreed with the DMSC prior to the analyses taking place. All data will be reported in accordance with the CONSORT statement. Baseline data will be summarised to check comparability between treatment arms, and screening data will be checked to highlight any characteristic differences between those individuals in the study, those ineligible, and those eligible but withholding consent.

The research team recognise the theoretical possibility of therapist effects. Here, we are randomising by individual; it is the same therapist who will be seeing people in both arms of the trial, and the treatment is individually delivered ensuring there are no group effects. To keep presentation of key information as standard throughout the study, the initial therapist contact will be described in the study manual and will use supporting media. We intend to recruit from a minimum of 30 sites where there will be multiple therapists taking part over the course of the study (e.g., sickness and holiday coverage). This gives the number of estimated therapist level clusters upwards of 60 and potential cluster sizes of around four participants in each arm of the trial. With such small cluster sizes, even if there were to be important therapist effects, their effect on statistical power would be minimal.

For peer review only - http://bmjopen.bmj.com/site/about/guidelines.xhtml

To address the possibility that these might exist, a single interim analysis is pre-planned to re-estimate the sample size. This will occur after approximately 200 participants have completed the three-month follow up questionnaire, whilst recruitment is still open. The pooled standard deviation of the primary outcome and presence of therapist effects will be estimated by calculating the therapist intraclass correlation coefficient and its 95% confidence interval (CI). Models will not include treatment effects. A revised sample size calculation will be discussed with DMC and TSC and if appropriate, permission requested from the funder to adjust the sample size.

All primary analyses are planned to be on an intention to treat basis with secondary per protocol analyses. The main analysis will investigate differences in the primary outcome measure, six months after randomisation, between the two treatment groups. Unadjusted and adjusted regression analyses will be used to estimate the between group difference. The adjusted analyses will adjust for the stratification variables, baseline scores and any other clinically important variables. Since individual clinicians will treat only a small number of participants enrolled in the trial, we do not expect clinician specific effects to be important in this study.

Descriptive statistics of functional outcome data (i.e. QuickDash and EQ-5D-5L) at each time point will be constructed with between group analyses following the method set out for the primary analysis above. Patterns of recovery will also be explored. Secondary analyses will include chi-squared tests to compare the number of dislocations and other complications between allocation groups. For important complications (e.g. dislocations), Kaplan Meier curves of the time to complication will be constructed. If sufficiently large numbers of complication groups are observed, Cox regression models will be constructed to compare the time to complication in each arm. Temporal effects will be investigated using a multi-level model of all follow up data.

Two pre-specified sub-group analyses will be undertaken to assess whether there is evidence of differing intervention effect: hand dominance (injured shoulder dominant arm *v* injured shoulder is non-dominant arm and age (younger participants *v* older participants). Because of the small sample size these analyses will be exploratory. The subgroup analyses will follow the methods described for the primary analysis, with additional interaction terms incorporated into the mixed-effects regression model to assess the level of support for these hypotheses. We will additionally present the main result separately for younger and older age groups for the benefit of future systematic reviewers who may be focused on different age groups. We will draw no inference from these analyses.

For peer review only - http://bmjopen.bmj.com/site/about/guidelines.xhtml

Careful monitoring of missingness and crossovers will be conducted. If judged appropriate, multiple imputation will be used to account for missing data, with all necessary assumptions reported. If large numbers of treatment cross-overs are observed, Complier-Average Causal Effect (CACE) models will be used.

RCT Health economic analysis

A prospectively planned analyses will be detailed in a health economic analysis plan (HEAP), agreed with the DMSC. Analysis will be conducted from an NHS and personal social services perspective, with methods adhering to the recommendations of the NICE Reference Case [13].

Costs of the intervention groups will be estimated, reflecting resource inputs associated with rehabilitation and broader healthcare resource utilisation. Resource use associated with the index condition will be captured through routine health service data collection systems and participant questionnaires administered at each follow up time point.

Unit costs will be estimated from local and national sources using established accounting methods, reflated to current prices. Health-related quality of life will be measured at baseline and at all follow time points using the EQ-5D-5L measure. Responses will be used to generate quality-adjusted life years (QALYs) using the UK value set recommended by NICE guidance at the time of analysis [14].

Within-trial analysis using bivariate regression of costs and QALYs, with multiple imputation of missing data, will inform a probabilistic assessment of incremental treatment cost-effectiveness. Missingness mechanisms will be explored and multiple imputation methods will be used where appropriate to avoid biases associated with complete case analysis. Findings will be analysed and visualised in the cost-effectiveness plane, as cost-effectiveness acceptability curves, net monetary benefit and value of information analysis. Costs and outcomes arising during the trial will be undiscounted, reflecting the 12-month time horizon. Sensitivity analyses will be undertaken to explore uncertainties and to consider issues of generalisability of the study.

SWAT analysis

For peer review only - http://bmjopen.bmj.com/site/about/guidelines.xhtml

All eligible participants will be included on an intention-to-treat basis, using two-sided statistical significance at the 5% level. We will summarise baseline characteristics of participants by the type of SWAT intervention sent. An average cost per participant will be estimated for each SWAT intervention arm. For the primary outcomes of questionnaire response rates, the difference in proportions will be calculated with 95% CIs, and the primary analysis will be a chi-squared test to assess statistical association. Additionally, a logistic regression adjusting for age, gender and host trial treatment allocation will be performed to investigate the effects of these variables. A per protocol analysis will also be performed.

The secondary outcome of time to questionnaire return will be assessed by a Kaplan Meier curve and the SWAT interventions compared by log rank test. Cox regression will be applied adjusting for participant age, gender and host trial treatment allocation, and the effect of the intervention reported. The requirement for any questionnaire return reminder will be analysed in the same way as the primary outcome).

Embedded Qualitative Study

Qualitative data will be analysed using the Framework method [15], which broadly includes data familiarisation; identifying a thematic framework; indexing; charting; mapping and interpretation. NVivo software will be used to facilitate this process. Researcher bias will be minimised through regular crosschecking of data and findings by members of the research team. Transcripts will be returned to participants (where necessary) providing them with the opportunity to check the transcripts for accuracy and authenticity and to offer any subsequent reflections. Quotes will be used as exemplars of key points in the writing up of these data. These qualitative data will be used to provide insight into participant's experiences of the interventions to provide context to the quantitative results.

Ethics and dissemination

This study was funded by NIHR HTA (16/167/56), 01/06/18; National Research Ethic Committee approved (18/WA/0236), 26/07/18. Registered on the International Standard Randomised Controlled Trial Number registry (ISRCTN63184243), 07/09/18. The ffirst site opened to recruitment 05/11/18. The SWAT was funded by MRC (MR/R013748/1), 01/05/19 and registered on the MRC-HTMR All-Ireland Hub (reference number SWAT 121).

For peer review only - http://bmjopen.bmj.com/site/about/guidelines.xhtml

The trial will be conducted in full conformance with the principles of the Declaration of Helsinki and to Good Clinical Practice guidelines and comply with all applicable UK legislation and Warwick University Standard Operating Procedures (SOPs). The trial will be reported in line with the CONSORT statement [16].

The results of this project will be disseminated through peer-reviewed journals, conference presentations amongst the orthopaedic and rehabilitation networks, policy makers, patient specific newsletters and local mechanisms at all participating centres.

Author contribution

RSK, MU, DE, EK, DT, SD, HB and CM wrote the background section and developed the research question. RSK, MU, DE, EK, DT, JB, ZL wrote the research methodology and management sections of the protocol. DE, NP, HP and JM wrote the sample size and analysis sections of the protocol. All authors reviewed and approved the final manuscript.

The research team would like to acknowledge Alwin McGibbon and Matt Kennedy for their contribution throughout the development and main trial as a PPI Representatives and Phil Moss for data clerk expertise.

Funding

This trial was funded by NIHR HTA (16/167/56), 01/06/18. The SWAT was supported by the Medical Research Council (MRC; grant number MR/R013748/1).

Disclaimer

The funder has not been involved in the design of the study. The views expressed are those of the authors and not necessarily those of NIHR or the Department of Health and Social Care.

Competing interests

RSK is a member of the UK NIHR HTA CET board, NIHR ICA Doctoral panel and previous member of the NIHR RfPB board. RSK, NP, DE, HP, JM, MU, SD, CM and DT have all been awarded current and previous NIHR research grants. JB, GD, ZL, AH,HN, EK and HB have none to declare.

MU has received travel expenses for speaking at conferences from the professional organisations hosting the conferences. He is a director and shareholder of Clinvivo Ltd that

For peer review only

provides electronic data collection for health services research. He is part of an academic partnership with Serco Ltd. He was an editor of the NIHR journal series for which he received a fee.

Data Statement

The data sharing plans for the current study will be updated via ISRCTN

Provenance and peer review

Not commissioned; peer reviewed for ethical and funding approval prior to submission.

References

1. Brownson, P., et al., *BESS/BOA Patient Care Pathways: Traumatic anterior shoulder instability.* Shoulder Elbow, 2015. **7**(3): p. 214-26.
2. Hanchard, N.C., L.M. Goodchild, and L. Kottam, *Conservative management following closed reduction of traumatic anterior dislocation of the shoulder.* Cochrane Database Syst Rev, 2014(4): p. CD004962.
3. Berendes, T.D., et al., *Survey on the management of acute first-time anterior shoulder dislocation amongst Dutch public hospitals.* Arch Orthop Trauma Surg, 2015. **135**(4): p. 447-54.
4. Dawson, J., R. Fitzpatrick, and A. Carr, *The assessment of shoulder instability. The development and validation of a questionnaire.* J Bone Joint Surg Br, 1999. **81**(3): p. 420-6.
5. Dawson, J., et al., *The Oxford shoulder score revisited.* Arch Orthop Trauma Surg, 2009. **129**(1): p. 119-23.
6. Gummesson, C., M.M. Ward, and I. Atroshi, *The shortened disabilities of the arm, shoulder and hand questionnaire (QuickDASH): validity and reliability based on responses within the full-length DASH.* BMC Musculoskelet Disord, 2006. **7**: p. 44.
7. Herdman, M., et al., *Development and preliminary testing of the new five-level version of EQ-5D (EQ-5D-5L).* Qual Life Res, 2011. **20**(10): p. 1727-36.
8. Moser, J.S., et al., *Comparison of two patient-based outcome measures for shoulder instability after nonoperative treatment.* J Shoulder Elbow Surg, 2008. **17**(6): p. 886-92.
9. van der Linde, J.A., et al., *The Oxford Shoulder Instability Score; validation in Dutch and first-time assessment of its smallest detectable change.* J Orthop Surg Res, 2015. **10**: p. 146.
10. Hoffmann, T.C., et al., *Better reporting of interventions: template for intervention description and replication (TIDieR) checklist and guide.* BMJ, 2014. **348**: p. g1687.
11. Slade, S.C., et al., *Consensus on Exercise Reporting Template (CERT): Modified Delphi Study.* Phys Ther, 2016. **96**(10): p. 1514-1524.
12. Mars, T., et al., *Fidelity in complex behaviour change interventions: a standardised approach to evaluate intervention integrity.* BMJ Open, 2013. **3**(11): p. e003555.
13. in *Guide to the Methods of Technology Appraisal 2013*. 2013: London.
14. NICE. *Position statement on use of the EQ-5D-5L value set for England (updated October 2019)*. 2019.
15. Ritchie J, L.J., *Qualitative research practice: a guide for social science students and researchers*. 2003, London: Sage.

For peer review only - http://bmjopen.bmj.com/site/about/guidelines.xhtml

16. Schulz, K.F., et al., *CONSORT 2010 statement: updated guidelines for reporting parallel group randomised trials.* BMJ, 2010. **340**: p. c332.

For peer review only

For peer review only - http://bmjopen.bmj.com/site/about/guidelines.xhtml

18 December 2017

NHS
National Institute for Health Research
Evaluation, Trials and Studies Coordinating Centre
University of Southampton
Alpha House
Enterprise Road
Southampton
SO16 7NS

Tel: 023 8059 5586

Email: netscomms@nihr.ac.uk
Web: www.nihr.ac.uk

Dr Rebecca Kearney
NIHR Clinical Lecturer
Clinical Trials Unit
The University of Warwick
Gibbet Hill Road
Coventry
West Midlands
CV4 7AL

Dear Dr Kearney

NIHR HTA Reference Number: 16/167/56
Acute Rehabilitation following Traumatic anterior shoulder dISlocAtioN (ARTISAN)- A Multi Centre RCT

I am pleased to inform you that the HTA Funding Board has recommended your application submitted for funding and the Department of Health, in their capacity as the National Institute for Health Research (NIHR), has confirmed their intention to award funding upon acceptance of the terms and conditions set out in the Standard Research Contract and pending agreement to the suggested amendments recommended by the Board, as detailed in the enclosures.

The Standard Research Contract, between Contractors and the Secretary of State for Health for all initiatives can be found on the NIHR website at:

https://www.nihr.ac.uk/funding-and-support/funding-for-research-studies/manage-my-study/contracts-and-intellectual-property.htm.

Next Steps

The NIHR is committed to the rapid initiation of research following the decision to fund to benefit patients as soon as possible. Therefore, we expect funded researchers to be working towards gaining the necessary contractual agreements and governance approvals required to start the project within 8 months or by a date mutually agreed by both parties on acceptance of the award.

The NIHR acknowledges the risk to organisations around committing resource to research before a contract is in place; however, it is rare to not reach contractual terms unless the circumstance of the research team changes. The NIHR, therefore, encourages organisations to commit staff to setting up projects at as early an opportunity as possible in order to expedite the formal commencement of research.

It is acknowledged that there can be unforeseen delays in starting up a research project, but in order to help reduce these it is your responsibility to work closely with your organisation's R&D department or equivalent as well as other colleagues / departments involved in the

The NIHR Evaluation, Trials and Studies Coordinating Centre (NETSCC), based at the University of Southampton, manages evaluation research programmes and activities for the NIHR

administration and management of the research, and to start these discussions at the earliest opportunity.

To ensure that the project starts within the agreed timeframe with all the required agreements and approvals in place, appropriate staff (such as project and/or study managers) need to be in post as early as possible after receiving this letter of intent. These staff costs will ultimately be covered through the research funding award, but you are encouraged to meet them from Research Capability Funding (RCF) or equivalent prior to the research contract being agreed.

To support the often-iterative process towards agreement of the contract, we have set out the timeframes for the submission of responses or information for each step towards the agreement of the Standard Research Contract as well as the anticipated start date.

- Responses to Board feedback and queries – no later than 5 weeks from the receipt of this letter.
- Responses to Finance and IP queries – 5 weeks from the receipt of this letter.
- Contract signature – no later than 7 months from the receipt of this letter.
- Contracted commencement start – no later than 8 months from the receipt of this letter or a date mutually agreed on acceptance of award.

On receipt of information as set out above, the NIHR through NETSCC is committed to responding to your submission of information within two weeks or we will update you on progress.

The enclosures to this letter detail the feedback on your application, the processes to be undertaken during the next steps, and additional information relating to your award. Your contact person within NETSCC is Rozz Bloom, who will be working with you on the contract.

Yours sincerely,

Lynn Kerridge
Chief Executive Officer, NETSCC

Enclosures:
- Board, Finance and IP Feedback
- Research Management and Approvals Information

cc: Dr Jane Prewett
University of Warwick
Jane.Prewett@warwick.ac.uk

Professor David Torgerson, Director York Trials Unit Department of Health Sciences Faculty of Sciences ARRC Building University Road, Heslington, York, YO10 5DD Tel: 01904 321340 Fax: 01904 321383 Email: david.torgerson@york.ac.uk
7th February 2019

Dear Ms Shah,

Re: PROMETHEUS SWAT award in the ARTISAN Trial

Thank you for your application for a PROMETHEUS award to undertake the following SWAT in the ARTISAN Trial:

(Hereinafter the "Host Trial")
'Do courtesy telephone calls or postcards to trial participants following enrolment increase future retention rates in the 'Acute Rehabilitation following Traumatic anterior shoulder dISlocAtioN' (ARTISAN) trial?' Ms A Shah, Ms J Brown, Dr H Parsons, Dr R Kearney. Warwick Clinical Trials Unit, University of Warwick, Coventry. CV4 7AL

(Hereinafter the "SWAT")

Following peer review by independent members of the PROMETHEUS programme team, I am pleased to inform you that your application to implement the SWAT in the Host Trial has been successful.

Value of award and award payments
The total value of your award is £5000 inclusive of any applicable VAT. This comprises: 30 hours of a senior statisticians time (Dr Helen Parsons); 100 hours of a data entry clerk's time (Phil Moss); £750 for Trial Manager, Amna Shah to attend MRC methodology conference and present results; and £725 for postage, printing and consumable costs.

The award period is from 01.05.2019 to 01.09.2021. Payment will be made in two instalments at the beginning of the SWAT recruitment period, and following receipt of the anonymised data, subject to the award holders supplying an invoice to the PROMETHEUS team.

Thank you for your application and congratulations on your award.

Please can you indicate your acceptance of this award and the terms and conditions listed below by returning one signed copy of this letter to Dr Adwoa Parker (adwoa.parker@york.ac.uk).

Ymchwil Iechyd a Gofal Cymru
Health and Care Research Wales

Gwasanaeth Moeseg Ymchwil
Research Ethics Service

Ariennir gan Lywodraeth Cymru
Funded by Welsh Government

Wales REC 3
Health and Care Research Wales Support and Delivery Centre
Castlebridge 4
15-19 Cowbridge Road East
Cardiff CF11 9AB

Telephone : 029 2078 5741
E-mail : helen.williams19@wales.nhs.uk
Website : www.hra.nhs.uk

Please note: This is the favourable opinion of the REC only and does not allow you to start your study at NHS sites in England until you receive HRA Approval

26 July 2018

Dr Rebecca Kearney
Associate Professor (Clinical)
University of Warwick
Warwick Clinical Trials Unit, Warwick Medical School
University of Warwick, Gibbett Hill Campus
Coventry
CV4 7AL

Dear Dr Kearney

**Study title:**	**Acute rehabilitation following traumatic anterior shoulder dislocation**
**REC reference:**	**18/WA/0236**
**IRAS project ID:**	**245863**

Thank you for your letter of 24 July 2018, responding to the Committee's request for further information on the above research and submitting revised documentation.

The further information has been considered on behalf of the Committee by the Chair.

We plan to publish your research summary wording for the above study on the HRA website, together with your contact details. Publication will be no earlier than three months from the date of this opinion letter. Should you wish to provide a substitute contact point, require further information, or wish to make a request to postpone publication, please contact hra.studyregistration@nhs.net outlining the reasons for your request.

Confirmation of ethical opinion
On behalf of the Committee, I am pleased to confirm a favourable ethical opinion for the above research on the basis described in the application form, protocol and supporting documentation as revised, subject to the conditions specified below.

Conditions of the favourable opinion
The REC favourable opinion is subject to the following conditions being met prior to the start of the study.

For peer review only

Management permission must be obtained from each host organisation prior to the start of the study at the site concerned.

Management permission should be sought from all NHS organisations involved in the study in accordance with NHS research governance arrangements. Each NHS organisation must confirm through the signing of agreements and/or other documents that it has given permission for the research to proceed (except where explicitly specified otherwise).
Guidance on applying for HRA and HCRW Approval (England and Wales)/ NHS permission for research is available in the Integrated Research Application System, at www.hra.nhs.uk or at http://www.rdforum.nhs.uk.

Where a NHS organisation's role in the study is limited to identifying and referring potential participants to research sites ("participant identification centre"), guidance should be sought from the R&D office on the information it requires to give permission for this activity.

For non-NHS sites, site management permission should be obtained in accordance with the procedures of the relevant host organisation.

Sponsors are not required to notify the Committee of management permissions from host organisations

Registration of Clinical Trials
All clinical trials (defined as the first four categories on the IRAS filter page) must be registered on a publically accessible database within 6 weeks of recruitment of the first participant (for medical device studies, within the timeline determined by the current registration and publication trees).

There is no requirement to separately notify the REC but you should do so at the earliest opportunity e.g. when submitting an amendment. We will audit the registration details as part of the annual progress reporting process.

To ensure transparency in research, we strongly recommend that all research is registered but for non-clinical trials this is not currently mandatory.

If a sponsor wishes to request a deferral for study registration within the required timeframe, they should contact hra.studyregistration@nhs.net. The expectation is that all clinical trials will be registered, however, in exceptional circumstances non registration may be permissible with prior agreement from the HRA. Guidance on where to register is provided on the HRA website.

It is the responsibility of the sponsor to ensure that all the conditions are complied with before the start of the study or its initiation at a particular site (as applicable).

Ethical review of research sites

NHS sites
The favourable opinion applies to all NHS sites taking part in the study, subject to management permission being obtained from the NHS/HSC R&D office prior to the start of the study (see "Conditions of the favourable opinion" below).

Non-NHS sites
The Committee has not yet completed any site-specific assessment (SSA) for the non-NHS research site(s) taking part in this study. The favourable opinion does not therefore apply to any non-NHS site at present. We will write to you again as soon as an SSA application(s) has been reviewed. In the meantime no study procedures should be initiated at non-NHS sites.

For peer review only

Approved documents
The final list of documents reviewed and approved by the Committee is as follows:

Document	*Version*	*Date*
Copies of advertisement materials for research participants [Poster]	1.0	26 June 2018
Evidence of Sponsor insurance or indemnity (non NHS Sponsors only) [Insurance Certificate]		
GP/consultant information sheets or letters [GP Letter]	1.2	18 June 2018
Interview schedules or topic guides for participants [Interview Schedule]	1.0	06 June 2018
IRAS Application Form [IRAS_Form_26062018]		26 June 2018
IRAS Application Form XML file [IRAS_Form_26062018]		26 June 2018
IRAS Checklist XML [Checklist_26062018]		26 June 2018
IRAS Checklist XML [Checklist_03072018]		03 July 2018
IRAS Checklist XML [Checklist_24072018]		24 July 2018
Letter from funder [Funding Confirmation Letter]		18 December 2017
Letter from sponsor [Sponsor Collaboration Agreement]		18 June 2018
Other [Screening Log]	1.2	18 June 2018
Other [Eligibility Checklist]	1.2	18 June 2018
Other [Consent Checklist]	1.3	18 June 2018
Other [Patient Contact Details Form]	1.2	18 June 2018
Other [Registration Form]	1.2	18 June 2018
Other [Pre-Injury Baseline Questionnaire]	1.3	18 June 2018
Other [Post-Injury Baseline Questionnaire]	1.4	18 June 2018
Other [6 Week Follow-Up Questionnaire]	0.2	18 June 2018
Other [3 Month Follow-Up Questionnaire]	0.2	18 June 2018
Other [6 Month Follow-Up Questionnaire]	1.3	18 June 2018
Other [12 Month Follow-Up Questionnaire]	0.2	18 June 2018
Other [SAE-Initial]	1.3	18 June 2018
Other [SAE-Follow-Up]	1.3	18 June 2018
Other [SAE-Continuation]	1.3	18 June 2018
Other [Notification of Death Form]	1.3	18 June 2018
Other [Post-Randomisation Withdrawal Form]	1.3	18 June 2018
Other [Post-Registration Withdrawal Form]	1.3	18 June 2018
Other [Site Delegation Log]	1.0	25 June 2018
Other [Interview Consent Form]	1.2	18 June 2018
Other [Interview Invitation Letter]	1.2	18 June 2018
Other [Interview Patient Information Sheet]	1.2	18 June 2018
Other [Collaboration Agreement with York CTU]		05 June 2018
Other [CI's Response to Provisional REC approval]	1.0	17 July 2018
Other [DMC Charter]	1.0	24 July 2018
Other [ARTISAN Summary Information Sheet]	1.0	19 July 2018
Participant consent form [Main Consent Form]	1.2	18 June 2018
Participant information sheet (PIS) [Main Patient Information Sheet]	1.2	18 June 2018
Participant information sheet (PIS) [Main Patient Information Sheet]	1.3	24 July 2018
Research protocol or project proposal [Protocol]	1.2	25 June 2018
Summary CV for Chief Investigator (CI) [Chief Investigator CV]	1.0	11 May 2018

For peer review only

Statement of compliance
The Committee is constituted in accordance with the Governance Arrangements for Research Ethics Committees and complies fully with the Standard Operating Procedures for Research Ethics Committees in the UK.

After ethical review
Reporting requirements

The attached document *"After ethical review – guidance for researchers"* gives detailed guidance on reporting requirements for studies with a favourable opinion, including:

- Notifying substantial amendments
- Adding new sites and investigators
- Notification of serious breaches of the protocol
- Progress and safety reports
- Notifying the end of the study

The HRA website also provides guidance on these topics, which is updated in the light of changes in reporting requirements or procedures.

User Feedback
The Health Research Authority is continually striving to provide a high quality service to all applicants and sponsors. You are invited to give your view of the service you have received and the application procedure. If you wish to make your views known please use the feedback form available on the HRA website: http://www.hra.nhs.uk/about-the-hra/governance/quality-assurance/

HRA Training
We are pleased to welcome researchers and R&D staff at our training days – see details at http://www.hra.nhs.uk/hra-training/

**18/WA/0236**	**Please quote this number on all correspondence**

With the Committee's best wishes for the success of this project.

Yours sincerely

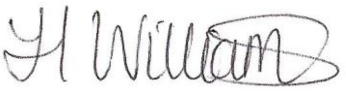

Mrs Helen Williams
Health and Care Research Wales
Research Ethics Committee Co-ordinator

pp Dr Pete Wall
Chair

Email - helen.williams19@wales.nhs.uk

Enclosures: "After ethical review – guidance for researchers" *[SL-AR2]*

Copy to: Mrs Jane Prewett
Ms Ceri Jones, University Hospital Coventry and Warwickshire

For peer review only

For peer review only

Reporting checklist for protocol of a clinical trial.

Based on the SPIRIT guidelines.

Instructions to authors

Complete this checklist by entering the page numbers from your manuscript where readers will find each of the items listed below.

Your article may not currently address all the items on the checklist. Please modify your text to include the missing information. If you are certain that an item does not apply, please write "n/a" and provide a short explanation.

Upload your completed checklist as an extra file when you submit to a journal.

In your methods section, say that you used the SPIRITreporting guidelines, and cite them as:

Chan A-W, Tetzlaff JM, Altman DG, Laupacis A, Gøtzsche PC, Krleža-Jerić K, Hróbjartsson A, Mann H, Dickersin K, Berlin J, Doré C, Parulekar W, Summerskill W, Groves T, Schulz K, Sox H, Rockhold FW, Rennie D, Moher D. SPIRIT 2013 Statement: Defining standard protocol items for clinical trials. Ann Intern Med. 2013;158(3):200-207

		Reporting Item	Page Number
**Administrative information**			
Title	#1	Descriptive title identifying the study design, population, interventions, and, if applicable, trial acronym	1

For peer review only

Trial registration	#2a	Trial identifier and registry name. If not yet registered, name of intended registry	3 & 16
Trial registration: data set	#2b	All items from the World Health Organization Trial Registration Data Set	3 &16
Protocol version	#3	Date and version identifier	3
Funding	#4	Sources and types of financial, material, and other support	3 & 16
Roles and responsibilities: contributorship	#5a	Names, affiliations, and roles of protocol contributors	17
Roles and responsibilities: sponsor contact information	#5b	Name and contact information for the trial sponsor	12
Roles and responsibilities: sponsor and funder	#5c	Role of study sponsor and funders, if any, in study design; collection, management, analysis, and interpretation of data; writing of the report; and the decision to submit the report for publication, including whether they will have ultimate authority over any of these activities	12 &17
Roles and responsibilities: committees	#5d	Composition, roles, and responsibilities of the coordinating centre, steering committee, endpoint adjudication committee, data	12

For peer review only

		management team, and other individuals or groups overseeing the trial, if applicable (see Item 21a for data monitoring committee)	
**Introduction**			
Background and rationale	#6a	Description of research question and justification for undertaking the trial, including summary of relevant studies (published and unpublished) examining benefits and harms for each intervention	3 & 4
Background and rationale: choice of comparators	#6b	Explanation for choice of comparators	3 & 4
Objectives	#7	Specific objectives or hypotheses	4, 5 & 7
Trial design	#8	Description of trial design including type of trial (eg, parallel group, crossover, factorial, single group), allocation ratio, and framework (eg, superiority, equivalence, non-inferiority, exploratory)	5
**Methods: Participants, interventions, and outcomes**			

For peer review only

Study setting	#9	Description of study settings (eg, community clinic, academic hospital) and list of countries where data will be collected. Reference to where list of study sites can be obtained	8
Eligibility criteria	#10	Inclusion and exclusion criteria for participants. If applicable, eligibility criteria for study centres and individuals who will perform the interventions (eg, surgeons, psychotherapists)	8
Interventions: description	#11a	Interventions for each group with sufficient detail to allow replication, including how and when they will be administered	9-11
Interventions: modifications	#11b	Criteria for discontinuing or modifying allocated interventions for a given trial participant (eg, drug dose change in response to harms, participant request, or improving / worsening disease)	9-11
Interventions: adherance	#11c	Strategies to improve adherence to intervention protocols, and any procedures for monitoring adherence (eg, drug tablet return; laboratory tests)	9-11
Interventions: concomitant care	#11d	Relevant concomitant care and interventions that are permitted or prohibited during the trial	9-11

For peer review only

Outcomes	#12	Primary, secondary, and other outcomes, including the specific measurement variable (eg, systolic blood pressure), analysis metric (eg, change from baseline, final value, time to event), method of aggregation (eg, median, proportion), and time point for each outcome. Explanation of the clinical relevance of chosen efficacy and harm outcomes is strongly recommended	6 & 7
Participant timeline	#13	Time schedule of enrolment, interventions (including any run-ins and washouts), assessments, and visits for participants. A schematic diagram is highly recommended (see Figure)	6
Sample size	#14	Estimated number of participants needed to achieve study objectives and how it was determined, including clinical and statistical assumptions supporting any sample size calculations	7 & 8
Recruitment	#15	Strategies for achieving adequate participant enrolment to reach target sample size	8

Methods: Assignment of interventions (for controlled trials)

For peer review only

Allocation: sequence generation	#16a	Method of generating the allocation sequence (eg, computer-generated random numbers), and list of any factors for stratification. To reduce predictability of a random sequence, details of any planned restriction (eg, blocking) should be provided in a separate document that is unavailable to those who enrol participants or assign interventions	8 & 9
Allocation concealment mechanism	#16b	Mechanism of implementing the allocation sequence (eg, central telephone; sequentially numbered, opaque, sealed envelopes), describing any steps to conceal the sequence until interventions are assigned	8 & 9
Allocation: implementation	#16c	Who will generate the allocation sequence, who will enrol participants, and who will assign participants to interventions	8 & 9
Blinding (masking)	#17a	Who will be blinded after assignment to interventions (eg, trial participants, care providers, outcome assessors, data analysts), and how	12
Blinding (masking): emergency unblinding	#17b	If blinded, circumstances under which unblinding is permissible, and procedure for revealing a participant's allocated intervention during the trial	12

For peer review only

Methods: Data collection, management, and analysis

Data collection plan	#18a	Plans for assessment and collection of outcome, baseline, and other trial data, including any related processes to promote data quality (eg, duplicate measurements, training of assessors) and a description of study instruments (eg, questionnaires, laboratory tests) along with their reliability and validity, if known. Reference to where data collection forms can be found, if not in the protocol	6 & 7
Data collection plan: retention	#18b	Plans to promote participant retention and complete follow-up, including list of any outcome data to be collected for participants who discontinue or deviate from intervention protocols	6, 7 & 9
Data management	#19	Plans for data entry, coding, security, and storage, including any related processes to promote data quality (eg, double data entry; range checks for data values). Reference to where details of data management procedures can be found, if not in the protocol	17

For peer review only

Statistics: outcomes	#20a	Statistical methods for analysing primary and secondary outcomes. Reference to where other details of the statistical analysis plan can be found, if not in the protocol	13-15
Statistics: additional analyses	#20b	Methods for any additional analyses (eg, subgroup and adjusted analyses)	13-15
Statistics: analysis population and missing data	#20c	Definition of analysis population relating to protocol non-adherence (eg, as randomised analysis), and any statistical methods to handle missing data (eg, multiple imputation)	13-15
**Methods: Monitoring**			
Data monitoring: formal committee	#21a	Composition of data monitoring committee (DMC); summary of its role and reporting structure; statement of whether it is independent from the sponsor and competing interests; and reference to where further details about its charter can be found, if not in the protocol. Alternatively, an explanation of why a DMC is not needed	12
Data monitoring: interim analysis	#21b	Description of any interim analyses and stopping guidelines, including who will have access to these interim results and make the final decision to terminate the trial	14

For peer review only

Harms	#22	Plans for collecting, assessing, reporting, and managing solicited and spontaneously reported adverse events and other unintended effects of trial interventions or trial conduct	12
Auditing	#23	Frequency and procedures for auditing trial conduct, if any, and whether the process will be independent from investigators and the sponsor	12
**Ethics and dissemination**			
Research ethics approval	#24	Plans for seeking research ethics committee / institutional review board (REC / IRB) approval	16 & 17
Protocol amendments	#25	Plans for communicating important protocol modifications (eg, changes to eligibility criteria, outcomes, analyses) to relevant parties (eg, investigators, REC / IRBs, trial participants, trial registries, journals, regulators)	16 & 17
Consent or assent	#26a	Who will obtain informed consent or assent from potential trial participants or authorised surrogates, and how (see Item 32)	8
6Consent or assent: ancillary studies	#26b	Additional consent provisions for collection and use of participant data and biological specimens in ancillary studies, if applicable	8

For peer review only

Confidentiality	#27	How personal information about potential and enrolled participants will be collected, shared, and maintained in order to protect confidentiality before, during, and after the trial	17
Declaration of interests	#28	Financial and other competing interests for principal investigators for the overall trial and each study site	17 & 18
Data access	#29	Statement of who will have access to the final trial dataset, and disclosure of contractual agreements that limit such access for investigators	18
Ancillary and post trial care	#30	Provisions, if any, for ancillary and post-trial care, and for compensation to those who suffer harm from trial participation	17
Dissemination policy: trial results	#31a	Plans for investigators and sponsor to communicate trial results to participants, healthcare professionals, the public, and other relevant groups (eg, via publication, reporting in results databases, or other data sharing arrangements), including any publication restrictions	16 & 17
Dissemination policy: authorship	#31b	Authorship eligibility guidelines and any intended use of professional writers	17

For peer review only

Dissemination policy: reproducible research	#31c	Plans, if any, for granting public access to the full protocol, participant-level dataset, and statistical code	18

Appendices

Informed consent materials	#32	Model consent form and other related documentation given to participants and authorised surrogates	Can be provided as supplementary material if requested.
Biological specimens	#33	Plans for collection, laboratory evaluation, and storage of biological specimens for genetic or molecular analysis in the current trial and for future use in ancillary studies, if applicable	8

None The SPIRIT checklist is distributed under the terms of the Creative Commons Attribution License CC-BY-ND 3.0. This checklist can be completed online using https://www.goodreports.org/, a tool made by the EQUATOR Network in collaboration with Penelope.ai

For peer review only

BMJ Open

Acute Rehabilitation following Traumatic anterior shoulder dISlocAtioN (ARTISAN): Protocol for a Multi Centre Randomised Controlled Trial

Journal:	*BMJ Open*
Manuscript ID	bmjopen-2020-040623.R1
Article Type:	Protocol
Date Submitted by the Author:	22-Sep-2020
Complete List of Authors:	Kearney, Rebecca; University of Warwick, Warwick Clinical Trials Unit<br>Dhanjal, Gurmit; University of Warwick, Warwick Clinical Trials Unit<br>Parsons, Nicholas; University of Warwick, Warwick Medical School<br>Ellard, David; University of Warwick, Clinical Trials Unit;<br>Parsons, Helen; University of Warwick, Division of Health Sciences<br>Haque, Aminul; University of Warwick, Warwick Clinical Trials Unit<br>Karasouli, Eleni; University of Warwick, Warwick Clinical Trials Unit<br>Mason, James; University of Warwick, Warwick Medical School<br>Nwankwo, Henry; University of Warwick, Warwick Clinical Trials Unit<br>Brown, Jaclyn; University of Warwick, Clinical Trials Unit; University of Warwick Clinical Sciences Research Institute, CTU<br>Liew, Zi Heng; University of Warwick, Warwick Clinical Trials Unit, Warwick Medical School<br>Drew, Stephen; University Hospitals Coventry and Warwickshire NHS Trust, Trauma and Orthopaedics<br>Modi, Chetan; University Hospitals Coventry and Warwickshire NHS Trust, Trauma and Orthopaedics<br>Bush, Howard; University Hospitals Coventry and Warwickshire NHS Trust, Trauma and Orthopaedics<br>Torgerson, David; The University of York,<br>Underwood, Martin; Warwick University, Warwick Medical School
**Primary Subject Heading**:	Rehabilitation medicine
Secondary Subject Heading:	Health economics, Research methods, Sports and exercise medicine
Keywords:	Shoulder < ORTHOPAEDIC & TRAUMA SURGERY, Rehabilitation medicine < INTERNAL MEDICINE, Clinical trials < THERAPEUTICS

SCHOLARONE™
Manuscripts

I, the Submitting Author has the right to grant and does grant on behalf of all authors of the Work (as defined in the below author licence), an exclusive licence and/or a non-exclusive licence for contributions from authors who are: i) UK Crown employees; ii) where BMJ has agreed a CC-BY licence shall apply, and/or iii) in accordance with the terms applicable for US Federal Government officers or employees acting as part of their official duties; on a worldwide, perpetual, irrevocable, royalty-free basis to BMJ Publishing Group Ltd ("BMJ") its licensees and where the relevant Journal is co-owned by BMJ to the co-owners of the Journal, to publish the Work in this journal and any other BMJ products and to exploit all rights, as set out in our licence.

The Submitting Author accepts and understands that any supply made under these terms is made by BMJ to the Submitting Author unless you are acting as an employee on behalf of your employer or a postgraduate student of an affiliated institution which is paying any applicable article publishing charge ("APC") for Open Access articles. Where the Submitting Author wishes to make the Work available on an Open Access basis (and intends to pay the relevant APC), the terms of reuse of such Open Access shall be governed by a Creative Commons licence – details of these licences and which Creative Commons licence will apply to this Work are set out in our licence referred to above.

Other than as permitted in any relevant BMJ Author's Self Archiving Policies, I confirm this Work has not been accepted for publication elsewhere, is not being considered for publication elsewhere and does not duplicate material already published. I confirm all authors consent to publication of this Work and authorise the granting of this licence.

For peer review only

TITLE PAGE

Title

Acute Rehabilitation following Traumatic anterior shoulder dISlocAtioN (ARTISAN): Protocol for a Multi Centre Randomised Controlled Trial

Corresponding author

Rebecca S Kearney: Professor, University of Warwick, Clinical Trials Unit, Gibbet Hill Road, Coventry, CV4 7AL & University Hospitals Coventry & Warwickshire NHS Trust, Trauma and Orthopaedics, Clifford Bridge Road, Coventry, CV2 2DX; R.S.Kearney@warwick.ac.uk; 02476 573156

Authors

- Gurmit Dhanjal: Trial Manager, University of Warwick, Warwick Medical School, Gibbet Hill Road, Coventry, CV4 7AL (Gurmit.Dhanjal@warwick.ac.uk)
- Nicholas Parsons: Associate Professor, University of Warwick, Warwick Medical School, Gibbet Hill Road, Coventry, CV4 7AL (Nick.Parsons@warwick.ac.uk)
- David Ellard: Associate Professor, University of Warwick, Clinical Trials Unit, Gibbet Hill Road, Coventry, CV4 7AL (D.R.Ellard@warwick.ac.uk)
- Helen Parsons: Senior Research Fellow, University of Warwick, Clinical Trials Unit, Gibbet Hill Road, Coventry, CV4 7AL (H.Parsons@warwick.ac.uk)
- Aminul Haque: Research Associate, University of Warwick, Clinical Trials Unit, Gibbet Hill Road, Coventry, CV4 7AL (Aminul.Haque.1@warwick.ac.uk)
- Eleni Karasouli: Senior Research Fellow, University of Warwick, Clinical Trials Unit, Gibbet Hill Road, Coventry, CV4 7AL (E.Karasouli@warwick.ac.uk)
- James Mason: Professor, University of Warwick, Clinical Trials Unit, Gibbet Hill Road, Coventry, CV4 7AL (J.Mason@warwick.ac.uk)
- Henry Nwankwo: Research Associate, University of Warwick, Clinical Trials Unit, Gibbet Hill Road, Coventry, CV4 7AL (Henry.Nwankwo@warwick.ac.uk)
- Jaclyn Brown: Deputy Head of Operations, University of Warwick, Clinical Trials Unit, Gibbet Hill Road, Coventry, CV4 7AL (J.Brown.10@warwick.ac.uk)

For peer review only

- ZiHeng Liew: Research Physiotherapist, University of Warwick, Clinical Trials Unit, Gibbet Hill Road, Coventry, CV4 7AL (Zi.Liew@warwick.ac.uk)
- Stephen Drew: Consultant Orthopaedic Surgeon, University Hospitals Coventry & Warwickshire NHS Trust, Trauma and Orthopaedics, Clifford Bridge Road, Coventry, CV2 2DX (Email: Steven.Drew@uhcw.nhs.uk)
- Chetan Modi: Consultant Orthopaedic Surgeon, University Hospitals Coventry & Warwickshire NHS Trust, Trauma and Orthopaedics, Clifford Bridge Road, Coventry, CV2 2DX (Email: Chetan.Modi@uhcw.nhs.uk)
- Howard Bush: Physiotherapist, University Hospitals Coventry & Warwickshire NHS Trust, Trauma and Orthopaedics, Clifford Bridge Road, Coventry, CV2 2DX (Email: Howard.Bush@uhcw.nhs.uk)
- David Torgerson: Professor, University of York, York Trials Unit, Department of Health Sciences, ARRC Building, Heslington, York, YO10 5DD (david.torgerson@york.ac.uk)
- Martin Underwood: Professor, University of Warwick, Clinical Trials Unit, Gibbet Hill Road, Coventry, CV4 7AL & University Hospitals Coventry & Warwickshire NHS Trust, Trauma and Orthopaedics, Clifford Bridge Road, Coventry, CV2 2DX (M.Underwood@warwick.ac.uk)

Word count: 4,055

ABSTRACT

Introduction

First time Traumatic Anterior Shoulder Dislocation (TASD) is predominantly managed non-operatively. People sustaining TASD have ongoing pain, disability, and future risk of re-dislocation. There are no published RCTs comparing different non-operative rehabilitation strategies to ascertain the optimum clinically effective approach after TASD.

Methods and analysis

In this multi-centre adaptive RCT, with internal pilot, adults with a radiologically confirmed first time TASD treated non-surgically will be screened at a minimum of 30 sites. People with neurovascular complications, bilateral dislocations, or are unable to attend physiotherapy will be excluded.

For peer review

For peer review only - http://bmjopen.bmj.com/site/about/guidelines.xhtml

Randomisation will be on a 1:1 treatment allocation, stratified by age, hand dominance and site. Participants will receive a single session of advice; or a single session of advice plus offer of further physiotherapy (maximum four months). The primary analysis will be the difference in Oxford Shoulder Instability Score (OSIS) at six months. A sample size of a minimum of 478 participants will allow us to show a four point difference with 90% power.

An embedded qualitative study will explore the participants' experiences of the trial interventions.

Ethics, registration and dissemination

Funded by NIHR HTA (16/167/56), 01/06/18; National Research Ethic Committee approved (18/WA/0236), 26/07/18. Registered on the International Standard Randomised Controlled Trial Number registry (ISRCTN63184243), 07/09/18. First site opened 05/11/18 and final results will be updated on trial registries and submitted to a peer-reviewed journal and will inform rehabilitation strategies after a TASD. SWAT funded by MRC (MR/R013748/1), 01/05/19; registered on the MRC-HTMR All-Ireland Hub (reference number SWAT 121).

Keywords

Shoulder dislocation, rehabilitation, physiotherapy, adaptive design

ARTICLE SUMMARY: STRENGHTS AND LIMITATIONS OF THIS STUDY

- UK wide trial across a minimum of 30 sites to optimise external validity.
- Primary outcome measure is patient-centred.
- The main limitation is that the intervention could not be blinded.

MAIN TEXT

Introduction

The shoulder is the most frequently dislocated joint; occurring in 8.2 to 23.9 per 100,000 people per year [1]. Traumatic Anterior Shoulder Dislocations (TASD) occur when excessive forces during a traumatic event displace the humeral head frontwards, out of the shoulder socket, resulting in the

joint surfaces completely losing contact [1-3]. They predominantly occur in men under 25 years during high impact events and women over 80 years during low impact events [1].

People sustaining TASD often have ongoing pain, disability, and morbidity linked to re-dislocation and subsequent need for repeated management [1-3]. Rehabilitation may reduce these events and restore a functional, painless shoulder through restoration of movement and retraining muscles [2]. However, a Cochrane review has found no evidence to support this [2]; Dutch national guidelines explicitly do not refer to physiotherapy [3]; and UK guidelines cite referral *'may be helpful'* [1].

This research protocol will investigate the hypothesis regarding the nature and extent of what physiotherapy is required for the management of patients following TASD with an embedded 'Study Within A Trial (SWAT)' to compare two strategies for retention of participants in RCTs.

Research questions

RCT Research question

In adults with first time TASD managed non-operatively does a single session of advice and course of physiotherapy improve the Oxford Shoulder Instability Score (OSIS) by a minimum of four additional points at six months when compared to a single session of advice only?

SWAT Research question

Does making a courtesy introductory telephone call to recruited participants improve response rates to six month follow-up questionnaires compared with a written card with similar information?

Objectives

RCT Primary objective

To quantify and draw inferences on observed differences in the OSIS score between a single session of advice and physiotherapy with a single session of advice only, for adults with first time TASD managed non-operatively, six months post randomisation.

For peer review only - http://bmjopen.bmj.com/site/about/guidelines.xhtml

RCT Secondary objectives

- To estimate comparative cost-effectiveness (cost/QALY) of the two trial treatments, from an NHS and personal social services perspective.
- To determine the difference in complication rate (e.g. shoulder re-dislocation) in the first 12 months between the trial treatment groups.
- To quantify and draw inferences between the functional status (OSIS) of the trial treatment groups at six weeks, three and 12 months.
- To quantify and draw inferences on observed differences in the functional status (QuickDASH) of between the trial treatment groups at six weeks, three, six and 12 months.
- To quantify and draw inferences on observed differences of health related quality of life (EQ-5D-5L) between the trial treatment groups at six weeks, three, six and 12 months.
- To qualitatively explore participants' experiences of receiving the trial treatments and identify facilitators and obstacles to adhering to them.

SWAT Primary objective

Evaluate the impact of making a courtesy call to newly recruited participants in the ARTISAN trial on response rates to six month follow-up questionnaires compared with a written card with similar information.

SWAT Secondary objectives

- Time to response to the questionnaires at each time points.
- Response rates at six weeks, three, six and twelve months.
- Completeness of responses.
- Number of reminder notices required.
- Cost of intervention per participant.

Methods and analysis

Study design

Multi-centre, adaptive, randomised controlled trial with internal pilot and SWAT

For peer review only - http://bmjopen.bmj.com/site/about/guidelines.xhtml

Outcome measures

Outcome measures will be collected at baseline (pre randomisation) at the recruiting site and then at six weeks, three, six and twelve months centrally from Warwick Clinical Trials Unit (WCTU) by postal questionnaire, mobile application, telephone or email.

RCT Primary outcome

OSIS: Self-completed outcome measure containing 12 questions (0-4 points each), with possible scores from 0 (best function) to 48 (worst function) [4, 5]. Questions relate to activities of daily living particularly relevant to patients exhibiting shoulder instability and has been designed to assess outcome of therapy.

RCT Secondary outcomes

QuickDASH: Self-completed shortened version of the Disabilities of the Arm, Shoulder and Hand (DASH) questionnaire. Uses 11 items to measure physical function and symptoms in people with any or multiple musculoskeletal disorders of the upper limb. Designed to help describe the disability experienced by people with upper-limb disorders and monitor changes in symptoms and function over time [6].

EQ-5D-5L: Health-related quality of life measure consisting of five dimensions, each with 5-levels of response. Each combination of answers is converted into a health utility score. Gives a single preference based index value for health status that can be used for broader cost-effectiveness comparative purposes [7].

Complications: Complications will be defined into three categories:
a) Pre-defined complications directly related to the interventions.
b) Pre-defined complications directly caused by the primary TASD event not identified by the initial assessing clinician, but subsequently identified.
c) Complications not related to the intervention or TASD event and will subsequently not be formally analysed or reported.

For peer review only - http://bmjopen.bmj.com/site/about/guidelines.xhtml

Resource use questionnaires: Primary health-economic analysis will concentrate on direct intervention and healthcare/personal social services costs, while wider impact (societal) costs will be included within the sensitivity analyses.

Embedded qualitative interviews: A purposive sample, up to 50 participants, informed by treatment allocation, gender, age, and outcome will be invited for one interview, to explore the participant experience of receiving the interventions and facilitators and obstacles to adhering to them.

SWAT Primary outcome

Response rate: Proportion of participants who return the questionnaire.

SWAT Secondary outcomes

Time to response: Date of first sending questionnaire to date of questionnaire received by study team.

Completeness of responses: Number of missing items per questionnaire.

Number of reminder notices: Number of notices administered by the trial team.

Cost of intervention: Cost of phone call or postcard per participant.

Sample size

RCT Sample size

A worthwhile difference from this intervention is 4 points on the OSIS [8, 9]. The standard deviation (SD) of the OSIS six months after injury is 10 points [8, 9]. However, the literature has predominantly included a young population, given that we will recruit a wider range of ages a larger SD is expected, therefore an SD of 12 is more likely. To show a four point difference at 5% significance level with 90% power allowing a margin of 20% loss during follow-up needs a minimum of 478 participants [5, 9].

For peer review only - http://bmjopen.bmj.com/site/about/guidelines.xhtml

SWAT Sample size

The sample size for the SWAT is limited by the host trial sample size. The SWAT opened to recruitment after the internal pilot (50 participants), therefore approximately 428 participants (214 per group) are expected and would provide 90% power and 5% significance to detect differences in retention rates of approximately 11%.

Screening and eligibility

RCT Screening and eligibility

Adults presenting at trial sites (NHS hospitals) with a first-time TASD, confirmed radiologically, managed non-operatively (decided by the treating clinician and patient) will be identified as potential participants. The site team will undertake eligibility checks and details will be entered on a monthly screening log.

Participants will be excluded if they have significant neurovascular complications; bilateral dislocations; have been randomised previously; unable to adhere to trial procedures, or unable attend physiotherapy within six weeks of injury.

All eligible participants will be provided with verbal and written information about the study. If eligible and willing to join the study, a consent form will be signed (Supplementary material one) followed by collection of baseline demographic data, pre and post injury functional outcomes.

SWAT screening and eligibility

Participants recruited into ARTISAN, who consent to being contacted by multiple methods, will be eligible for the SWAT.

Randomisation

RCT Randomisation

For peer review only - http://bmjopen.bmj.com/site/about/guidelines.xhtml

Participants will be randomised at the end of the initial advice session via a web based system. Allocation concealment will be maintained by an independent randomisation team at WCTU, who will be responsible for generation of the sequence and will have no role in the allocation of participants. The treatment group will be allocated by computer using a minimisation algorithm with a random element and stratification by participant age (39 years old and under and 40 years old and over), hand dominance and recruiting site.

SWAT randomisation

Minimisation with a random factor will be used to avoid imbalance between the SWAT intervention arms. The allocation ratio will be 1:1 and stratified by the ARTISAN allocation arm. Allocation concealment will be maintained by an independent randomisation team who will be responsible for generation of the sequence and have no role in the allocation of participants.

Post randomisation withdrawals

Participants may discontinue at any time without prejudice. Unless a participant explicitly withdraws consent, data will be collected until the end of the trial. For participants explicitly withdrawing consent for follow up procedures, trial data obtained up until the point of withdrawal will be included. Participants will have the option to withdraw from the trial-related questionnaires, but continue to provide routine NHS data for trial purposes. Participants who withdraw will not be replaced. Participants may be withdrawn from the trial at the discretion of the investigator and/or Trial Steering Committee for safety concerns.

Interventions

RCT Interventions

The interventions will be reported in line with the TIDIER and CERT checklists [10, 11]. All participating centres will receive an initial training session from the trial team. Following this, a lead physiotherapist at each site will be identified to complete subsequent training of additional physiotherapists. This training will be supported with web-based materials and a trial intervention manual.

For peer review only - http://bmjopen.bmj.com/site/about/guidelines.xhtml

All participants will receive a period of initial immobilisation in internal rotation as per UK national guidelines, for a duration of up to two weeks from date of injury [1]. At this time participants will be provided with a web-link to Phase 1 of the ARTISAN advice materials and provided with a paper based booklet version covering:

- What has happened to me?
- What can go wrong?
- How do I stop this happening again?
- How long do I have to wear my sling?
- Should I move my arm?
- How do I control my pain?
- When can I return to usual activities?
- What if something goes wrong?

All participants will subsequently receive an appointment for a physiotherapy advice session within six weeks of injury. At the first appointment, all participants will receive a single session of advice to aid self-management, lasting up to one hour and administered by an ARTISAN trained physiotherapist. Following routine assessment, the physiotherapist will deliver the above core set of intervention components and in addition:

- Points of contact if complications occur or expected recovery times are not achieved.
- A core set of progressive phase 2 range of movement exercises and what they aim to achieve.
- Enhancing self-management behaviours through the addition of goal setting, exercise planning and diaries.

The physiotherapist will provide details of web based materials, which will include all the core components above in written and video format, and will include a dedicated area for participants to set goals and keep diaries. The physiotherapist will also inform the participants that the website resources contain phase 3 progressive strengthening exercises and what they aim to achieve and later stage phase 4 progression to aid return to sports. Participants will be offered paper based alternatives.

For peer review only - http://bmjopen.bmj.com/site/about/guidelines.xhtml

Following completion of the first physiotherapy appointment the participant will be randomised, allocating them to this advice session alone or to this advice session plus the offer of additional physiotherapy.

Participants randomised to the advice session only, will only receive this physiotherapy intervention. However, if they are experiencing ongoing issues which are not improving, as part of the control intervention, the advice is for the participant to contact the clinic they attended or see their GP. If a participant follows this advice and self-initiates further physiotherapy this will not be deemed a deviation from the protocol. However, if someone other than the participant initiates further physiotherapy (another clinician) then this will be deemed a protocol deviation. This data will be collected as part of the trial dataset.

Participants randomised to receive additional physiotherapy will be offered of at least one additional physiotherapy session. Each additional session will last for up to 30 minutes, over a maximum duration of four months from date of randomisation. The number and frequency of any additional sessions will be jointly agreed between the clinician and participant, in keeping with standard practice. The course of physiotherapy will involve teaching and supervising the 'core set' of progressive exercises offered to the control arm in addition to being able to tailor treatment as per their usual practice.

SWAT Interventions

Participants will be randomised to receive either:

1) An introductory call within two weeks of being randomised to include thanking participants; reminding them of their valuable contribution and that they will be contacted at six weeks and three, six and twelve months; informing them of when the trial results are expected and to contact the ARTISAN team if they have queries.

2) A postcard-sized hand written card, with similar content as above, signed by the Chief Investigator and Trial Manager, posted to participants within one week of being randomised.

Fidelity and quality assurance of interventions

For peer review only - http://bmjopen.bmj.com/site/about/guidelines.xhtml

The trial team will implement a standardised approach of evaluating fidelity [12] of direct observations and audio recordings (twice annually); and self-reporting of the trial interventions. Any issues will be discussed by the trial management group (TMG), who will recommend appropriate action such as feedback to sites and additional training. If issues are not resolved following recommendations they will be escalated to oversight committees.

During site quality assurance visits the research teams will also be asked to provide radiological confirmation of the participants initial shoulder dislocation. If evidence is not found, participants will be withdrawn from the main trial sample, but will continue follow up procures and retained as part of a pre-planned sensitivity analysis.

Blinding

As the type of rehabilitation used will be clear to the participant, they cannot be blind to treatment. The treating clinician will also not be blind, but will take no part in follow up processes, these will be managed centrally from the Warwick CTU to reduce the assessment bias.

Adverse event management

Serious adverse events (SAEs) will be entered onto the SAE reporting form and reported to the central study team. SAEs that may be expected will be pre-defined and recorded on the participant's follow up questionnaire. SAEs that may be expected as part of a TASD are: damage to nerve or blood vessels, fractures, re-dislocation, torn ligaments or muscles, persistent exacerbation of shoulder pain, restriction of range of movement, adhesive capsulitis (frozen shoulder) and persistent instability. All SAEs will be reported to the sponsor (Warwick University and University Hospitals Coventry and Warwickshire), ethics committee and oversight committees.

End of trial

The trial will end when all participants have completed their 12-month follow-up. The trial will be stopped prematurely if mandated by the Ethics Committee; following recommendations from relevant oversight committees; or funding ceases.

Trial oversight

For peer review only - http://bmjopen.bmj.com/site/about/guidelines.xhtml

The TMG will meet monthly and report to the independent Trail Steering Committee (TSC) and Data Monitoring and Safety Committee (DMSC). The TSC and DMSC will hold meetings annually and be responsible for approval of the protocol, monitoring and supervising progress.

Patient and Public Involvement (PPI)

The clinical team consulted with patients when developing this project and subsequently we had a meeting with a group representing our target participants. We discussed experiences and expectations of services and plans for the trial. Service users' perspectives were key in the development of this protocol. PPI membership is represented on TMG and TSC committees and have reviewed all study materials. PPI members of the team will contribute to dissemination on trial completion.

Analysis

RCT Statistical Analysis

The internal pilot will inform and test recruitment strategies for the main trial in 12 sites over six months. A recruitment rate of one participant per month per centre was estimated for the six month pilot phase, with 50 participants in total. The decision to progress to the main trial will be made based on pre-defined progression criteria. The criteria for continuing will be achievement of 75-100% of the recruitment target; criteria for the need to review and amend trial procedures before continuing will be achieving 50-75% of the target; criteria for not proceeding will be achieving less than 50% of the target. These randomised participants will be retained in the final analysis.

A detailed statistical analysis plan (SAP) which will be agreed with the DMSC prior to the analyses taking place. All data will be reported in accordance with the CONSORT statement. Baseline data will be summarised to check comparability between treatment arms, and screening data will be checked to highlight any characteristic differences between those individuals in the study, those ineligible, and those eligible but withholding consent.

The research team recognise the theoretical possibility of therapist effects. Here, we are randomising by individual; it is the same therapist who will be seeing people in both arms of the trial, and the treatment is individually delivered ensuring there are no group effects. To keep

For peer review only - http://bmjopen.bmj.com/site/about/guidelines.xhtml

presentation of key information as standard throughout the study, the initial therapist contact will be described in the study manual and will use supporting media. We intend to recruit from a minimum of 30 sites where there will be multiple therapists taking part over the course of the study (e.g., sickness and holiday coverage). This gives the number of estimated therapist level clusters upwards of 60 and potential cluster sizes of around four participants in each arm of the trial. With such small cluster sizes, even if there were to be important therapist effects, their effect on statistical power would be minimal.

To address the possibility that these might exist, a single interim analysis is pre-planned to re-estimate the sample size. This will occur after approximately 200 participants have completed the three-month follow up questionnaire, whilst recruitment is still open. The pooled standard deviation of the primary outcome and presence of therapist effects will be estimated by calculating the therapist intraclass correlation coefficient and its 95% confidence interval (CI). Models will not include treatment effects. A revised sample size calculation will be discussed with DMC and TSC and if appropriate, permission requested from the funder to adjust the sample size.

All primary analyses are planned to be on an intention to treat basis with secondary per protocol analyses. The main analysis will investigate differences in the primary outcome measure, six months after randomisation, between the two treatment groups. Unadjusted and adjusted regression analyses will be used to estimate the between group difference. The adjusted analyses will adjust for the stratification variables, baseline scores and any other clinically important variables. Since individual clinicians will treat only a small number of participants enrolled in the trial, we do not expect clinician specific effects to be important in this study.

Descriptive statistics of functional outcome data (i.e. QuickDash and EQ-5D-5L) at each time point will be constructed with between group analyses following the method set out for the primary analysis above. Patterns of recovery will also be explored. Secondary analyses will include chi-squared tests to compare the number of dislocations and other complications between allocation groups. For important complications (e.g. dislocations), Kaplan Meier curves of the time to complication will be constructed. If sufficiently large numbers of complication groups are observed, Cox regression models will be constructed to compare the time to complication in each arm. Temporal effects will be investigated using a multi-level model of all follow up data.

For peer review only - http://bmjopen.bmj.com/site/about/guidelines.xhtml

Two pre-specified sub-group analyses will be undertaken to assess whether there is evidence of differing intervention effect: hand dominance (injured shoulder dominant arm *v* injured shoulder is non-dominant arm and age (younger participants *v* older participants). Because of the small sample size these analyses will be exploratory. The subgroup analyses will follow the methods described for the primary analysis, with additional interaction terms incorporated into the mixed-effects regression model to assess the level of support for these hypotheses. We will additionally present the main result separately for younger and older age groups for the benefit of future systematic reviewers who may be focused on different age groups. We will draw no inference from these analyses.

Careful monitoring of missingness and crossovers will be conducted. If judged appropriate, multiple imputation will be used to account for missing data, with all necessary assumptions reported. If large numbers of treatment cross-overs are observed, Complier-Average Causal Effect (CACE) models will be used.

RCT Health economic analysis

A prospectively planned analyses will be detailed in a health economic analysis plan (HEAP), agreed with the DMSC. Analysis will be conducted from an NHS and personal social services perspective, with methods adhering to the recommendations of the NICE Reference Case [13].

Costs of the intervention groups will be estimated, reflecting resource inputs associated with rehabilitation and broader healthcare resource utilisation. Resource use associated with the index condition will be captured through routine health service data collection systems and participant questionnaires administered at each follow up time point.

Unit costs will be estimated from local and national sources using established accounting methods, reflated to current prices. Health-related quality of life will be measured at baseline and at all follow time points using the EQ-5D-5L measure. Responses will be used to generate quality-adjusted life years (QALYs) using the UK value set recommended by NICE guidance at the time of analysis [14].

Within-trial analysis using bivariate regression of costs and QALYs, with multiple imputation of missing data, will inform a probabilistic assessment of incremental treatment cost-effectiveness. Missingness mechanisms will be explored and multiple imputation methods will be used where

For peer review only - http://bmjopen.bmj.com/site/about/guidelines.xhtml

appropriate to avoid biases associated with complete case analysis. Findings will be analysed and visualised in the cost-effectiveness plane, as cost-effectiveness acceptability curves, net monetary benefit and value of information analysis. Costs and outcomes arising during the trial will be undiscounted, reflecting the 12-month time horizon. Sensitivity analyses will be undertaken to explore uncertainties and to consider issues of generalisability of the study.

SWAT analysis

All eligible participants will be included on an intention-to-treat basis, using two-sided statistical significance at the 5% level. We will summarise baseline characteristics of participants by the type of SWAT intervention sent. An average cost per participant will be estimated for each SWAT intervention arm. For the primary outcomes of questionnaire response rates, the difference in proportions will be calculated with 95% CIs, and the primary analysis will be a chi-squared test to assess statistical association. Additionally, a logistic regression adjusting for age, gender and host trial treatment allocation will be performed to investigate the effects of these variables. A per protocol analysis will also be performed.

The secondary outcome of time to questionnaire return will be assessed by a Kaplan Meier curve and the SWAT interventions compared by log rank test. Cox regression will be applied adjusting for participant age, gender and host trial treatment allocation, and the effect of the intervention reported. The requirement for any questionnaire return reminder will be analysed in the same way as the primary outcome).

Embedded Qualitative Study

Qualitative data will be analysed using the Framework method [15], which broadly includes data familiarisation; identifying a thematic framework; indexing; charting; mapping and interpretation. NVivo software will be used to facilitate this process. Researcher bias will be minimised through regular crosschecking of data and findings by members of the research team. Transcripts will be returned to participants (where necessary) providing them with the opportunity to check the transcripts for accuracy and authenticity and to offer any subsequent reflections. Quotes will be used as exemplars of key points in the writing up of these data. These qualitative data will be used to provide insight into participant’s experiences of the interventions to provide context to the quantitative results.

For peer review only - http://bmjopen.bmj.com/site/about/guidelines.xhtml

Ethics and dissemination

This study was funded by NIHR HTA (16/167/56), 01/06/18; National Research Ethic Committee approved (18/WA/0236), 26/07/18. Registered on the International Standard Randomised Controlled Trial Number registry (ISRCTN63184243), 07/09/18. The ffirst site opened to recruitment 05/11/18. The SWAT was funded by MRC (MR/R013748/1), 01/05/19 and registered on the MRC-HTMR All-Ireland Hub (reference number SWAT 121).

The trial will be conducted in full conformance with the principles of the Declaration of Helsinki and to Good Clinical Practice guidelines and comply with all applicable UK legislation and Warwick University Standard Operating Procedures (SOPs). The trial will be reported in line with the CONSORT statement [16].

The results of this project will be disseminated through peer-reviewed journals, conference presentations amongst the orthopaedic and rehabilitation networks, policy makers, patient specific newsletters and local mechanisms at all participating centres.

Main strengths and limitations

This is the first UK wide multi centre RCT across a minimum of NHS 30 sites. The primary outcome measure is patient-centred. The main limitation is that the intervention could not be blinded.

Author contribution

RSK, MU, DE, EK, DT, SD, HB and CM wrote the background section and developed the research question. RSK, MU, DE, EK, DT, JB, ZL, GD wrote the research methodology and management sections of the protocol. DE, NP, HP, AH and JM, HN wrote the sample size and analysis sections of the protocol. All authors reviewed and approved the final manuscript.

Acknowledgement

The research team would like to acknowledge Alwin McGibbon and Matt Kennedy for their contribution throughout the development and main trial as a PPI Representatives and Phil Moss for data clerk expertise.

Funding

For peer review only - http://bmjopen.bmj.com/site/about/guidelines.xhtml

This trial was funded by NIHR HTA (16/167/56), 01/06/18. The SWAT was supported by the Medical Research Council (MRC; grant number MR/R013748/1).

Disclaimer

The funder has not been involved in the design of the study. The views expressed are those of the authors and not necessarily those of NIHR or the Department of Health and Social Care.

Competing interests

RSK is a member of the UK NIHR HTA CET board, NIHR ICA Doctoral panel and previous member of the NIHR RfPB board. RSK, NP, DE, HP, JM, MU, SD, CM and DT have all been awarded current and previous NIHR research grants. JB, GD, ZL, AH,HN, EK and HB have none to declare.

MU has received travel expenses for speaking at conferences from the professional organisations hosting the conferences. He is a director and shareholder of Clinvivo Ltd that provides electronic data collection for health services research. He is part of an academic partnership with Serco Ltd. He was an editor of the NIHR journal series for which he received a fee.

Data Statement

The data sharing plans for the current study will be updated via ISRCTN

Provenance and peer review

Not commissioned; peer reviewed for ethical and funding approval prior to submission.

References

1. Brownson, P., et al., *BESS/BOA Patient Care Pathways: Traumatic anterior shoulder instability.* Shoulder Elbow, 2015. **7**(3): p. 214-26.
2. Hanchard, N.C., L.M. Goodchild, and L. Kottam, *Conservative management following closed reduction of traumatic anterior dislocation of the shoulder.* Cochrane Database Syst Rev, 2014(4): p. CD004962.
3. Berendes, T.D., et al., *Survey on the management of acute first-time anterior shoulder dislocation amongst Dutch public hospitals.* Arch Orthop Trauma Surg, 2015. **135**(4): p. 447-54.
4. Dawson, J., R. Fitzpatrick, and A. Carr, *The assessment of shoulder instability. The development and validation of a questionnaire.* J Bone Joint Surg Br, 1999. **81**(3): p. 420-6.

For peer review only - http://bmjopen.bmj.com/site/about/guidelines.xhtml

5. Dawson, J., et al., *The Oxford shoulder score revisited.* Arch Orthop Trauma Surg, 2009. **129**(1): p. 119-23.
6. Gummesson, C., M.M. Ward, and I. Atroshi, *The shortened disabilities of the arm, shoulder and hand questionnaire (QuickDASH): validity and reliability based on responses within the full-length DASH.* BMC Musculoskelet Disord, 2006. **7**: p. 44.
7. Herdman, M., et al., *Development and preliminary testing of the new five-level version of EQ-5D (EQ-5D-5L).* Qual Life Res, 2011. **20**(10): p. 1727-36.
8. Moser, J.S., et al., *Comparison of two patient-based outcome measures for shoulder instability after nonoperative treatment.* J Shoulder Elbow Surg, 2008. **17**(6): p. 886-92.
9. van der Linde, J.A., et al., *The Oxford Shoulder Instability Score; validation in Dutch and first-time assessment of its smallest detectable change.* J Orthop Surg Res, 2015. **10**: p. 146.
10. Hoffmann, T.C., et al., *Better reporting of interventions: template for intervention description and replication (TIDieR) checklist and guide.* BMJ, 2014. **348**: p. g1687.
11. Slade, S.C., et al., *Consensus on Exercise Reporting Template (CERT): Modified Delphi Study.* Phys Ther, 2016. **96**(10): p. 1514-1524.
12. Mars, T., et al., *Fidelity in complex behaviour change interventions: a standardised approach to evaluate intervention integrity.* BMJ Open, 2013. **3**(11): p. e003555.
13. in *Guide to the Methods of Technology Appraisal 2013*. 2013: London.
14. NICE. *Position statement on use of the EQ-5D-5L value set for England (updated October 2019)*. 2019.
15. Ritchie J, L.J., *Qualitative research practice: a guide for social science students and researchers*. 2003, London: Sage.
16. Schulz, K.F., et al., *CONSORT 2010 statement: updated guidelines for reporting parallel group randomised trials.* BMJ, 2010. **340**: p. c332.

For peer review only - http://bmjopen.bmj.com/site/about/guidelines.xhtml

WARWICK CLINICAL TRIALS UNIT

York Trials Unit

<<INSERT LOCAL TRUST LOGO>>

NHS University Hospitals Coventry and Warwickshire NHS Trust

ARTISAN

Participant ID: | | | | |

2. Main Consent Form

Please read each statement carefully and initial the box if you agree — **Please *initial* box**

1. I confirm that I have read and understood the information sheet (Version ___ Date ______) for the ARTISAN study. I have had the opportunity to consider the information, ask questions and have had these answered satisfactorily. ☐
2. I understand that my participation is voluntary and that I am free to withdraw at any time, without giving any reason, without my medical care or legal rights being affected. ☐
3. I understand that relevant sections of any of my medical notes and data collected during the study may be looked at by responsible individuals at regulatory authorities or from the NHS Trust, where it is relevant to my taking part in this research. I give permission for these individuals to have access to my records. ☐
4. I understand that appropriate personal identifying information will be collected, stored and used by Warwick Clinical Trials Unit to enable follow up of my health status. This is on the understanding that any information will be treated with the strictest security and confidentiality. ☐
5. I understand that the information held and maintained by the Health and Social Care Information Centre and other Central UK NHS bodies may be used to help contact me or provide information about my health status. ☐
6. I understand the information collected about me will be used to support other research in the future, and may be shared anonymously with other researchers. ☐
7. I agree to my GP being informed of my participation in the study. ☐
8. I agree to take part in the above study. ☐

The below are not mandatory but please let us know your preference.

9. I agree to being contacted about future research related to my shoulder dislocation. Yes ☐ No ☐

10a. I agree to use the ARTISAN mobile App for follow-up data collection - if NO see 10b Yes ☐ No ☐

10b. *If you have opted out of using the ARTISAN mobile app for follow up (10a), the method of follow-up will be via postal questionnaires, do you consent to text message reminders by the ARTSAN study* Yes ☐ No ☐

11. I agree to my session being observed and audio recorded to monitor quality. Yes ☐ No ☐

Patient Name	Signature	Date (DD/MMM/YYYY)
Name of person taking consent	Signature	Date (DD/MMM/YYYY)

Original copy to be kept in the ARTISAN site file, one copy to be given to the patient and one copy to be kept in the participants medical notes.

This project is funded by the NIHR HTA programme (Ref: 16/167/56).

FUNDED BY NIHR | National Institute for Health Research

For peer review only - http://bmjopen.bmj.com/site/about/guidelines.xhtml

Reporting checklist for protocol of a clinical trial.

Based on the SPIRIT guidelines.

Instructions to authors

Complete this checklist by entering the page numbers from your manuscript where readers will find each of the items listed below.

Your article may not currently address all the items on the checklist. Please modify your text to include the missing information. If you are certain that an item does not apply, please write "n/a" and provide a short explanation.

Upload your completed checklist as an extra file when you submit to a journal.

In your methods section, say that you used the SPIRITreporting guidelines, and cite them as:

Chan A-W, Tetzlaff JM, Altman DG, Laupacis A, Gøtzsche PC, Krleža-Jerić K, Hróbjartsson A, Mann H, Dickersin K, Berlin J, Doré C, Parulekar W, Summerskill W, Groves T, Schulz K, Sox H, Rockhold FW, Rennie D, Moher D. SPIRIT 2013 Statement: Defining standard protocol items for clinical trials. Ann Intern Med. 2013;158(3):200-207

		Reporting Item	Page Number
**Administrative information**			
Title	#1	Descriptive title identifying the study design, population, interventions, and, if applicable, trial acronym	1

For peer review only

Trial registration	#2a	Trial identifier and registry name. If not yet registered, name of intended registry	3 & 16
Trial registration: data set	#2b	All items from the World Health Organization Trial Registration Data Set	3 &16
Protocol version	#3	Date and version identifier	3
Funding	#4	Sources and types of financial, material, and other support	3 & 16
Roles and responsibilities: contributorship	#5a	Names, affiliations, and roles of protocol contributors	17
Roles and responsibilities: sponsor contact information	#5b	Name and contact information for the trial sponsor	12
Roles and responsibilities: sponsor and funder	#5c	Role of study sponsor and funders, if any, in study design; collection, management, analysis, and interpretation of data; writing of the report; and the decision to submit the report for publication, including whether they will have ultimate authority over any of these activities	12 &17
Roles and responsibilities: committees	#5d	Composition, roles, and responsibilities of the coordinating centre, steering committee, endpoint adjudication committee, data	12

For peer review only

		management team, and other individuals or groups overseeing the trial, if applicable (see Item 21a for data monitoring committee)	
**Introduction**			
Background and rationale	#6a	Description of research question and justification for undertaking the trial, including summary of relevant studies (published and unpublished) examining benefits and harms for each intervention	3 & 4
Background and rationale: choice of comparators	#6b	Explanation for choice of comparators	3 & 4
Objectives	#7	Specific objectives or hypotheses	4, 5 & 7
Trial design	#8	Description of trial design including type of trial (eg, parallel group, crossover, factorial, single group), allocation ratio, and framework (eg, superiority, equivalence, non-inferiority, exploratory)	5
**Methods: Participants, interventions, and outcomes**			

For peer review only

Study setting	#9	Description of study settings (eg, community clinic, academic hospital) and list of countries where data will be collected. Reference to where list of study sites can be obtained	8
Eligibility criteria	#10	Inclusion and exclusion criteria for participants. If applicable, eligibility criteria for study centres and individuals who will perform the interventions (eg, surgeons, psychotherapists)	8
Interventions: description	#11a	Interventions for each group with sufficient detail to allow replication, including how and when they will be administered	9-11
Interventions: modifications	#11b	Criteria for discontinuing or modifying allocated interventions for a given trial participant (eg, drug dose change in response to harms, participant request, or improving / worsening disease)	9-11
Interventions: adherance	#11c	Strategies to improve adherence to intervention protocols, and any procedures for monitoring adherence (eg, drug tablet return; laboratory tests)	9-11
Interventions: concomitant care	#11d	Relevant concomitant care and interventions that are permitted or prohibited during the trial	9-11

For peer review only

Outcomes	#12	Primary, secondary, and other outcomes, including the specific measurement variable (eg, systolic blood pressure), analysis metric (eg, change from baseline, final value, time to event), method of aggregation (eg, median, proportion), and time point for each outcome. Explanation of the clinical relevance of chosen efficacy and harm outcomes is strongly recommended	6 & 7
Participant timeline	#13	Time schedule of enrolment, interventions (including any run-ins and washouts), assessments, and visits for participants. A schematic diagram is highly recommended (see Figure)	6
Sample size	#14	Estimated number of participants needed to achieve study objectives and how it was determined, including clinical and statistical assumptions supporting any sample size calculations	7 & 8
Recruitment	#15	Strategies for achieving adequate participant enrolment to reach target sample size	8

Methods: Assignment of interventions (for controlled trials)

For peer review only

Allocation: sequence generation	#16a	Method of generating the allocation sequence (eg, computer-generated random numbers), and list of any factors for stratification. To reduce predictability of a random sequence, details of any planned restriction (eg, blocking) should be provided in a separate document that is unavailable to those who enrol participants or assign interventions	8 & 9
Allocation concealment mechanism	#16b	Mechanism of implementing the allocation sequence (eg, central telephone; sequentially numbered, opaque, sealed envelopes), describing any steps to conceal the sequence until interventions are assigned	8 & 9
Allocation: implementation	#16c	Who will generate the allocation sequence, who will enrol participants, and who will assign participants to interventions	8 & 9
Blinding (masking)	#17a	Who will be blinded after assignment to interventions (eg, trial participants, care providers, outcome assessors, data analysts), and how	12
Blinding (masking): emergency unblinding	#17b	If blinded, circumstances under which unblinding is permissible, and procedure for revealing a participant's allocated intervention during the trial	12

For peer review only

Methods: Data collection, management, and analysis

Data collection plan	#18a	Plans for assessment and collection of outcome, baseline, and other trial data, including any related processes to promote data quality (eg, duplicate measurements, training of assessors) and a description of study instruments (eg, questionnaires, laboratory tests) along with their reliability and validity, if known. Reference to where data collection forms can be found, if not in the protocol	6 & 7
Data collection plan: retention	#18b	Plans to promote participant retention and complete follow-up, including list of any outcome data to be collected for participants who discontinue or deviate from intervention protocols	6, 7 & 9
Data management	#19	Plans for data entry, coding, security, and storage, including any related processes to promote data quality (eg, double data entry; range checks for data values). Reference to where details of data management procedures can be found, if not in the protocol	17

For peer review only

Statistics: outcomes	#20a	Statistical methods for analysing primary and secondary outcomes. Reference to where other details of the statistical analysis plan can be found, if not in the protocol	13-15
Statistics: additional analyses	#20b	Methods for any additional analyses (eg, subgroup and adjusted analyses)	13-15
Statistics: analysis population and missing data	#20c	Definition of analysis population relating to protocol non-adherence (eg, as randomised analysis), and any statistical methods to handle missing data (eg, multiple imputation)	13-15
**Methods: Monitoring**			
Data monitoring: formal committee	#21a	Composition of data monitoring committee (DMC); summary of its role and reporting structure; statement of whether it is independent from the sponsor and competing interests; and reference to where further details about its charter can be found, if not in the protocol. Alternatively, an explanation of why a DMC is not needed	12
Data monitoring: interim analysis	#21b	Description of any interim analyses and stopping guidelines, including who will have access to these interim results and make the final decision to terminate the trial	14

For peer review only

Harms	#22	Plans for collecting, assessing, reporting, and managing solicited and spontaneously reported adverse events and other unintended effects of trial interventions or trial conduct	12
Auditing	#23	Frequency and procedures for auditing trial conduct, if any, and whether the process will be independent from investigators and the sponsor	12
**Ethics and dissemination**			
Research ethics approval	#24	Plans for seeking research ethics committee / institutional review board (REC / IRB) approval	16 & 17
Protocol amendments	#25	Plans for communicating important protocol modifications (eg, changes to eligibility criteria, outcomes, analyses) to relevant parties (eg, investigators, REC / IRBs, trial participants, trial registries, journals, regulators)	16 & 17
Consent or assent	#26a	Who will obtain informed consent or assent from potential trial participants or authorised surrogates, and how (see Item 32)	8
6Consent or assent: ancillary studies	#26b	Additional consent provisions for collection and use of participant data and biological specimens in ancillary studies, if applicable	8

For peer review only

Confidentiality	#27	How personal information about potential and enrolled participants will be collected, shared, and maintained in order to protect confidentiality before, during, and after the trial	17
Declaration of interests	#28	Financial and other competing interests for principal investigators for the overall trial and each study site	17 & 18
Data access	#29	Statement of who will have access to the final trial dataset, and disclosure of contractual agreements that limit such access for investigators	18
Ancillary and post trial care	#30	Provisions, if any, for ancillary and post-trial care, and for compensation to those who suffer harm from trial participation	17
Dissemination policy: trial results	#31a	Plans for investigators and sponsor to communicate trial results to participants, healthcare professionals, the public, and other relevant groups (eg, via publication, reporting in results databases, or other data sharing arrangements), including any publication restrictions	16 & 17
Dissemination policy: authorship	#31b	Authorship eligibility guidelines and any intended use of professional writers	17

For peer review only

Dissemination policy: reproducible research	#31c	Plans, if any, for granting public access to the full protocol, participant-level dataset, and statistical code	18

Appendices

Informed consent materials	#32	Model consent form and other related documentation given to participants and authorised surrogates	Can be provided as supplementary material if requested.
Biological specimens	#33	Plans for collection, laboratory evaluation, and storage of biological specimens for genetic or molecular analysis in the current trial and for future use in ancillary studies, if applicable	8

None The SPIRIT checklist is distributed under the terms of the Creative Commons Attribution License CC-BY-ND 3.0. This checklist can be completed online using https://www.goodreports.org/, a tool made by the EQUATOR Network in collaboration with Penelope.ai

For peer review only
